# Using Ionic Liquids to Improve CO_2_ Capture

**DOI:** 10.3390/molecules29225388

**Published:** 2024-11-15

**Authors:** Francisco Jose Alguacil, Jose Ignacio Robla

**Affiliations:** Centro Nacional de Investigaciones Metalurgicas (CSIC), Avda. Gregorio del Amo 8, 28040 Madrid, Spain; fjalgua@cenim.csic.es

**Keywords:** CO_2_ capture, absorption, adsorption, membranes, environment, global warming

## Abstract

Most of our energy consumption proceeds from the use of fossil fuels and the production of natural gas. However, the presence of impurities in this gas, like CO_2_, makes treatment necessary to avoid further concerns, such as greenhouse gas emissions, the corrosion of industrial equipment, etc.; thus, the development of CO_2_ capture and storage procedures is of the utmost importance in order to decrease CO_2_ production and mitigate its contribution to global warming. Among the CO_2_ capture processes available, three separation technologies are being used to achieve this goal: absorption, adsorption and membranes. To overcome some limitations of these methodologies, the joint use of these technologies with ionic liquids is gaining interest. The present work reviewed the most recent developments (for 2024) in CO_2_ capture using ionic liquids coupled to absorption-, adsorption- or membrane-based processes.

## 1. Introduction

A key factor in reducing our dependency on fossil fuels and, at the same time, improving/maintaining energy efficiencies is the reduction in CO_2_ emissions, or at least reducing the emissions of this gas into the atmosphere using so-called carbon capture and storage technologies.

CO_2_ capture, especially through chemical absorption using an aqueous amine-based solvent, is considered the most established and effective approach for the removal of this gas [1]. However, this technology presents some drawbacks, which are normally related to water content in the solvent that makes it difficult for solvent regeneration [2,3]. With their supporters and detractors, adsorption and membrane technologies are also considered as alternative procedures to the above in order to remove CO_2_ from gas streams.

These three technologies have a trait in common, which is that they can use ionic liquids (ILs) to improve their performance in the capture of CO_2_. ILs are a type of chemical that have been considered green solvents due to their properties. This consideration is due to their low vapor pressure (low volatility), high thermal stability and non-flammability. While ILs are formed exclusively by ions in liquid form at temperatures below 100 °C, one characteristic presented by these ILs is the possibility of tunability or modification of the cation or anion forming the salt in order to meet specific applications. In ILs, the cation and anion can have an organic or inorganic nature, and whereas the cation is commonly formed by an organic moiety, the anion is formed by organic or inorganic compounds. The appealing properties of ionic liquids make them interesting eco-friendly candidates for capturing CO_2_ from natural gas.

Some odd points regarding ILs are related to their high viscosity; at the same time, there is some controversy about the “green” nature of ILs [4,5,6,7].

In addition to their potential uses in cleaning gases, ILs have been considered as promising solvents, with special properties in organic synthesis, catalysis, electrochemistry, separation of metals, biomass processing, pharmaceuticals, tribology and energy storage devices, such as batteries, supercapacitors, fuel cells, etc. [8,9,10,11,12].

Aimed at CO_2_ capture, some of the recent literature reviewed the utilization of ionic liquid-functionalized metal–organic frameworks [13,14], different types of ashes [15], polymeric ionic liquids [16], ionic liquid-membrane-based systems [17], the application of machine learning techniques [18], ionic liquid-based advanced porous organic hyper-crosslinked polymers [19] and the general use of ionic liquids in CO_2_ adsorption and absorption procedures [20,21,22,23,24,25,26].

The present work presents further data (for 2024) about the applications of absorption, adsorption and membrane technologies coupled with the use of ionic liquids in CO_2_ removal or capture from gas streams. Also, recent studies in the literature on the use of ionic liquid-based aqueous biphasic solvents in this field are presented.

## 2. Absorption and Ionic Liquids

In absorption-based processing, CO_2_ is removed from the syngas stream before the hydrogen purification stage occurs. Ionic liquids are a type of sustainable and efficient CO_2_ absorbent because of their apparent advantages in providing a flexible alternative to standard absorbents such as amine solutions.

### 2.1. Ionic Liquids in Monophasic Systems

Studies on the process modelling of traditional and conventional amines, i.e., monoethanolamine and methyldietahanolamine, are very limited; thus, a packed column was used to derive the profiles of the operational variables affecting CO_2_ loading onto the diethylenetriamine (DETA) solution [18]. The process model was validated using experimental data from the literature. Furthermore, the CO_2_ absorption efficiency of the DETA solution was compared with that of the ionic liquid 1-n-butyl-3-methylimidazolium hexafluorophosphate ([BMIM][PF6]). Though the CO_2_ absorption efficiency of the ionic liquid was higher than that of the DETA solution, under the same operational conditions, the apparent disadvantages of the former (higher viscosity and price) should be further considered without compromising solvent performance.

The next work investigated the use of deep learning models for CO_2_ solubility prediction in ionic liquids using a dataset comprising 10,116 carbon dioxide solubility data points in 164 types of ionic liquids at various temperatures and pressures [19]. Deep neural network models, including an artificial neural network (ANN) and long short-term memory (LSTM), were developed to predict CO_2_ solubility in the ionic liquids. The ANN and LSTM models had convenient test accuracy in predicting CO_2_ solubility, with R^2^ values of 0.986 and 0.985, respectively. The ANN model presented reliable accuracy with a lower computational time (approximately 30-times faster) than the LSTM model.

A 2D computational fluid dynamic (CFD) model in COMSOL Multiphysics^®^ was developed to investigate CO_2_ absorption into ionic liquid 1-butyl-3-methylimidazolium tricyanomethanide ([BMIM][TCM]) [20]. Under various experimental conditions, a quadratic model for absorption (*p*-value < 0.0001 and R^2^ > 0.98) was presented. The maximum CO_2_ concentration was obtained at a pressure of 18.26 bar, temperature of 36 °C, and velocity of 0.825 m/s, whereas the sparger radius to column diameter was 0.414, and column height to diameter was 2.5.

The next work investigated CO_2_ capture, storage and conversion using the integration of plasma-ionic liquid technology [21]. 1-butyl-3-methylimidazolium chloride was utilized to capture and store CO_2_ under atmospheric pressure; further, plasma was used to assist in the reduction of removed CO_2_ into CO.

Since many investigations regarding CO_2_ absorption in ionic liquids are based on the use of imidazolium-based reagents, there is a lack of comprehensive understanding of how the different factors that govern the gas solubility affect the capture of this molecule onto these chemicals. Thus, an automated high-throughput setup for the measurement of CO_2_ solubility in ionic liquids combining six different anions and nine different cations (Table 1), forming up to 19 different specific ranges of compounds, was used [22]. Among all the ionic liquids investigated, the maximum void fraction and minimum electrostatic interaction were for [BMIM][DCA] and [BMPYRR][Triflate], respectively, and the minimum Henry’s constant, corresponding to maximum CO_2_ removal, was obtained using [BMP][NTF_2_]. CO_2_ solubility was better in compounds having fluorinated anions, especially when considering identical cations.

The role of the active hydrogen atom in the ionic liquid anion is a key point to understand the performance of these chemicals for CO_2_ capture; thus, the physical properties, CO_2_ capture and conversion of three hydantoin-based anion-functionalized ionic liquids ([P_4442_][Hy], [P_4442_]_2_[Hy], and [HDBU][Hy]) were investigated [23]. The results showed that the active hydrogen atom in the anion formed anionic hydrogen bonding networks, resulting in an increase in the melting point and viscosity and decreasing the basicity of the ionic liquid, losing their properties for CO_2_ capture and conversion. Whereas [P_4442_]_2_[Hy] shows better carbon dioxide capture and conversion performance attributable to the double-site interaction, [HDBU][Hy] presented the weakest catalytic CO_2_ conversion due to its anion and cation having active hydrogen atoms. The reference reported that the active hydrogen atom in the anion played a predominant role in the properties and potential applications of ionic liquids than the active hydrogen atom in the cation.

The next reference investigated the geometry and structural parameters of interaction of CO_2_ with several ionic liquids using state-of-the-art quantum chemical tools [24]. A maximum of up to eight CO_2_ molecules were computationally found to be possible to interact with the corresponding ionic liquid. The molecular electrostatic potential map (MESP) analysis revealed that the nucleophilicity of [Suc] moiety is decreased upon an increase in nCO_2_ molecules, while the electrophilicity of [P_4442_][Suc] increased with an increase in CO_2_ uptake. Moreover, the nature of non-covalent interactions existing between the CO_2_ molecules and [P4442][Suc] anion was also investigated using non-covalent interaction (NCI) and quantum theory of atoms in molecules (QTAIM) analysis.

The authors of [25] described the fabrication procedures of imidazolium cyanopyrrolide for electrochemical studies in consideration of precursor composition and reaction time. The ionic liquids and different diluents (i.e., acetonitrile, dimethylformamide, dimethyl sulfoxide, propylene carbonate, and n-methyl-2-pyrrolidone) were used to investigate the influence on conductivity, CO_2_ mass transport, and electrocatalytic reduction of CO_2_ (ECO2R) activation overpotential, together with the effects of electrode materials (Sn, Ag, Au, and glassy carbon). Acetonitrile was found to be the best diluent to decrease the onset potential and to enhance the catalytic current density for the production of CO, which is attributable to the increase in the ion mobility in combination with the silver electrode. The ECO2R activity of molecular catalysts Ni(cyclam)Cl_2_ and iron tetraphenylsulfonato porphyrin (FeTPPS) on the carbon cloth electrode was responsible for the high Faradaic efficiencies for CO in the presence of the ionic liquid.

Functionalized ionic liquids (IL-Br, IL-BF_4_, and IL-PF_6_) were formulated and characterized with a number of analytical tools [26]. These ionic liquids presented an efficient removal of CO_2_ from natural gas at ambient temperature. IL-PF_6_ was the formulation with the highest CO_2_ uptake capacity from the natural gas stream, with efficiencies up to 94% in 5 min. It was experimentally found that each gram of IL-PF_6_, IL-BF_4_, and IL-Br captured 2.02, 1.23, and 1.18 mmol of CO_2_, respectively. These efficiencies depended on the flow rate, being maximum at the lowest value (0.7 sccm). They concluded that CO_2_ is captured by the ionic liquids via the amine mechanism, where the lone pair of amino groups of the ionic liquid attacks the CO_2_ molecule. Another ionic liquid molecule is protonated by attracting the proton of the amine-reacted CO_2_, and a bimolecular compound of ionic liquid containing one carbon dioxide molecule is formed. The functionalized ionic liquids utilized in the investigation contained 3-(2-aminopropyl)-2-ethyl-1-propyl-1H-imidazol-3-ium as a cationic moiety and bromide, tetrafluoroborate or hexafluorophosphate as anions for the respective IL-Br, IL-BF_4_ and IL-PF_6_ ionic liquids.

Following the tendency of the formulation of new ionic liquids, three ionic liquids formed by tailoring an imidazolium cation with amino acid anions were synthesized and characterized [27]. The performance of propane–sultone-modified imidazolium cation [1MeimPS]^+^ and amino acid anions (tryptophan ([Trp]^−^), tyrosine ([Tyr]^−^), and histidine ([His]^−^)) on CO_2_ uptake was investigated at 25 °C and pressures up to 4 MPa. Of the above, [1MeimPS][His] had the best results in terms of CO_2_ absorption (Table 2) at different pressures. Moreover, a direct association between CO_2_ capacity and pressure increase was observed. The atoms in molecules theory (QTAIM) and Interaction Region Indicator (IRI) analysis revealed that the CO_2_ mainly interacted with anions through hydrogen bonding and van der Waal forces. The natural bond orbital (NBO) analysis showed that stable charge transfers occurred from CO_2_ towards the ionic liquids.

Adapted from [27]. x_2_ is defined as

(1) $$x_{2}=\frac{n_{2}^{liq}}{n_{1}^{liq}+n_{2}^{liq}}$$

where n_1_^liq^ is the number of CO_2_ moles in the feed gas and n_2_^liq^ is the number of moles of CO_2_ adsorbed onto the ionic liquids.

A machine learning framework was developed to predict the density, viscosity, and heat capacity of a series of anion-functionalized ionic liquids for CO_2_ capture, especially those with tetraalkylphosphonium cations and aprotic N-heterocyclic anions (AHAs) [28]. Various feature sets were considered using group contribution-based (GC) descriptors and descriptors extracted from COSMO-RS sigma profiles (SP) to build Support Vector Regression (SVR) and Gradient-Boosted Regression (GBR) machine learning models. The best fit for viscosity used GC-based descriptors and the SVR model, achieving a test set %AARD of 12.5% and R^2^ of 0.989. Density was modeled using these same descriptors with the SVR model framework and was fitted with a test set %AARD of 1.0%.

The absorption kinetics of CO_2_ into aqueous amino-acid-functionalized monoethanolamine glycinate ionic liquid in a stirred cell reactor was investigated [29]. The use of amino acids was convenient because these chemicals are less volatile and present good resistance against oxidative degradation. Experiments were carried out in a temperature range of 25–35 °C and solution concentrations ranging from 0.5 to 2.5 M in the pseudo-first-order regime. In order to check the fast pseudo-first-order kinetic model assumption, the dimensionless Hatta number and infinite enhancement factor were calculated. Increasing the ionic liquid concentration and temperature resulted in an increase in the reaction rate. The obtained kinetic constants have the same order of magnitude as those existing for primary amines. The reaction between the ionic liquids and CO_2_ is explained by the zwitterion formation (CH_2_COONH_2_^+^COO^−^), which acted as an intermediate product that is deprotonated by a base (water, amine, hydroxyl) to form carbamate:

(2) $$CH_{2}COONH_{2}^{+}COO^{-}+base\to {CH}_{2}COONHCOO^{-}+baseH^{+}$$

The authors of [30] presented data about the absorption mechanisms of carbon dioxide (also hydrogen sulfide and methane) for two imidazolium-based ionic liquids, 1-ethyl-3-methylimidazolium thiocyanate ([EMIM][SCN]) and 1-ethyl-3-methylimidazolium dicyanamide ([EMIM][DCA]). The results reveal that CO_2_ presented an important affinity for the interface over the bulk in both ionic liquids. Density profiles showed CO_2_′s higher density in [EMIM][DCA] compared to [EMIM][SCN], demonstrating the influence of anionic composition on CO_2_ solubility.

A thermodynamic model based on the cubic plus association equation of state was utilized to calculate the contribution from the chemical reactions between various stoichiometric ratios of CO_2_ and amino acid-ionic liquid (AAIL) (Nm:n, m:n is the stoichiometric ratio) on CO_2_ removal [31]. The results show that the larger contribution to the CO_2_ removal in [Glu], [Tyr] and [Gln]-ionic liquids, with strong polar groups, was attributable to the association scheme N1:2. The N1:1 association interaction was the dominant contribution of [Gly], [Ala], [Val] and [Pro]-ionic liquids, with hydrogen atom or short alkyl side chains, and [VBIM]-ionic liquid with multiple amino groups. The N2:1 association interaction was primary between CO_2_ and [Cho][His] or [Cho][Arg]. In addition, the contributions of these association effects were greater than that of dispersion and repulsion to CO_2_ solubility. More amino groups resulted in higher regeneration energy consumption and did not increase absorbent cyclic capacity. Among the studied AAILs, [Cho][Gly], which exhibited high cyclic capacity and low energy consumption, had the best potential towards CO_2_ capture.

To operate under typical flue gas stream conditions (8–10% CO_2_, 18–20% H_2_O, 2–3% O_2_, and 67–72% N_2_), one study modeled the combinations of aprotic heterocyclic anion-type ionic liquids (AHA-IL) with monoethanolamine (MEA) and investigated the carbon capture effectiveness of these mixtures [32]. All AHA-IL/MEA mixtures exhibited 32% lower reaction enthalpy (compared to MEA) and 64% lower viscosity (compared to AHA-IL), improving the properties towards gas capture with respect to amines and a series of ionic liquids. However, mixtures of the ionic liquids and non-aqueous amine presented an outlet stream temperature exceeding the degradation temperature of the amine (>120 °C) due to the exothermic character of CO_2_ capture on these systems. Against this, systems containing aqueous amine solutions did not present this characteristic, since water acted as a coolant, absorbing the excess heat. It is concluded that only the use of solvents consisting of the ionic liquids and aqueous amine solutions are suitable for CO_2_ capture operations.

A ferritin-tagged CA variant (DvCA8.0-F) was used in order to investigate its performance for CO_2_ capture [33]. After 14 weeks of incubation in a 50% MDEA solution at 50 °C, DvCA8.0-F maintained activity nearly equivalent to the initial one. The results show that DvCA8.0-F reduced the absorption time from 70 to 50 min at 40 °C and desorption time from 40 to 25 min at 96 °C in 25%MDEA+1%[N_1111_][Gly] (tetramethylammonium glycinate) solution, with a final CO_2_ upload and a stripped amount of CO_2_ at the same time. DvCA8.0-F exhibits the maximum reaction rate at 96 °C. When the concentration of DvCA8.0-F was 1.5 g/L, the absorption rate of CO_2_ reached about 90% of that of the 25% MEA solution.

Computational frameworks can both facilitate and improve the process of finding convenient ionic liquids via accurate property predictions in a data-driven manner. The next reference [34] used a framework consisting of two essential steps: establishing a correlation between the ionic liquid viscosity and CO_2_ solubility by joining two deep learning models (DNN-GC and ANN-GC) and using this correlation to identify the optimal ionic liquid to yield maximum CO_2_ uptake capacity. The algorithm generated novel ionic liquids, 1-decyl-2,3-dimethylimidazolium thiocyanate, 1-nonyl-3-methylimidazolium dicyanamide, and octyltrimethylammonium bis(trifluoromethyl sulfonyl)imide, predicting viscosity and CO_2_ solubility at pre-specified conditions (20 °C and 1.5 MPa).

With the goal to create new systems to be used in the removal of CO_2_ from gas streams, the well-known amine extractant, MDEA, was mixed with ionic liquids in order to formulate hybrid solvents and investigated its performance against this gas removal. Thus, in this work, 30 wt% MDEA was mixed with imidazolium-based ionic liquids in formulations of 5, 10 15 and 20 wt% [35]. The ILs were 1-butyl-3-methylimidazolium dicyanamide [BMIM][DCA], 1-butyl-3-methylimidazolium acetate [BMIM][Ac] and 1-butyl-3-methylimidazolium trifluoromethanesulfonate [BMIM][TfO]. The temperature had a negligible influence on the viscosity above 65 °C, whereas the CO_2_ uptake onto the different hybrid systems and the single amine followed the order [BMIM][TfO] + MDEA > [BMIM][DCA] + MDEA > [BMIM][Ac] + MDEA > MDEA. Experimental results indicated that the optimum formulation for CO_2_ removal was 30 wt% amine and 10 wt% ionic liquid. The system 30 wt% MDEA + 10 wt% [BMIM][TfO] presented fast CO_2_ absorption kinetics, whereas the highest absorption rate was reached at 50 bar.

In the next reference [36], different advanced intelligent models, Extreme Gradient Boosting (XGBoost), Gradient Boosting (GBoost), Light Gradient Boosting Machine (LightGBM), and Categorical Boosting (CatBoost) were used to predict the solubility of CO_2_ in 160 different ionic liquids. This prediction was based on variables such as the temperature, pressure, and chemical structure of the ionic liquids. The results indicate that the XGBoost model presented the most accurate results, with root mean square error (RMSE) and R^2^ values of 0.014 and 0.9967, respectively. It is determined that increasing pressure, decreasing temperature, and lengthening the alkyl chain resulted in an increase in the solubility of CO_2_ in the ionic liquids. Moreover, pressure and the number of –CH_2_ substituents in the ionic liquid have the most significant impact on the CO_2_ capture in these compounds.

A number of dicationic ionic liquids composed of di(tetraalkylphosphonium) cations with tributyl chains and carbon chains ranging from propyl to undecyl, with 2-cyanopyrrolide acting as the counter-anion, presented an improved thermal stability if compared to their monocationic aprotic-heterocyclic anion ionic liquid (AHA IL) equivalents [37]. The dicationic ionic liquids all have similar carbon dioxide loading per anion, although they exhibit an intermediate capacity between the presented monocationic ionic liquids and 2-cyanopyrrolide anion and that of tetraalkylphosphonium cations with longer (trihexyl(tetradecyl)) and shorter (triethyloctyl) chains. The presence of oxygen resulted in the formation of phosphine oxides (generic formulation R_3_PO), both in monocationic and dicationic ionic liquids.

An amino-functionalized ionic liquid [G1.0 PAMAM] [2-MI] combined with water was used in CO_2_ capture [38]. The ionic liquid was formed by amine groups belonging to generation 1.0 of dendrimers (G1.0 PAMAM) and 2-methylimidazole (2-MI). The experimental results showed that 42.71 wt% [G1.0 PAMAM] [2-MI]/H_2_O had an uptake of 6.81 mol CO_2_/mol (38.5° C, aeration rate 100 mL/min), which was much higher than that of MEA/H_2_O. With the combined effect of 2-MI and water, the viscosities of the solution before and after absorption at 40 °C were 35.3 and 53.8 mPa·s, respectively, which were lower than the values presented by most of the ionic liquid. The reaction mechanism of [G1.0 PAMAM] [2-MI] with CO_2_ was attributed to a synergistic combination of a zwitterionic mechanism and a base-catalyzed hydration mechanism to produce carbamate (primary and secondary amine and 2-MI) or carbonate/bicarbonate (tertiary amines).

Superbase-derived ionic liquids (SILs) are absorbents that improve the carbon challenge featured by tunable interaction strength with CO_2_ via structural engineering, particularly the oxygenate-derived counterparts (i.e., phenolate). In spite of the above, there are still some concerns about the usefulness of these compounds due to some issues related to their stability. To resolve these doubts, the next reference developed pyrazolonate-derived SILs, possessing an anti-oxidation nature, by introducing aza-fused rings in the oxygenate-derived anions, enhancing CO_2_ uptake capacity via a carbonate formation pathway (O−C bond formation) [39].

Ionic liquids, 1-decyl-3-methylimidazolium bis(trifluromethylsulfonyl) imide [IL1], 1-hexadecyl-3-methylimidazolium bis(trifluromethylsulfonyl) imide) [IL2] and triethytetradecylammonium bis(trifluromethylsulfonyl) imide [IL3], were used to investigate their performance on CO_2_ capture [40]. Solubility experiments were conducted in the 30−70 °C temperature range, with pressures up to 1.5 MPa. The results show that [IL2] is the best candidate for CO_2_ capture, which is attributable to its longer alkyl chain.

This reference investigates the interactions of three amino acid anions (glycine [Gly], histidine [His], and arginine [Arg]) with the cation 1-methoxylbutyl-3-methylimidazolium [MOBMIM] and their performance in the capture of CO_2_ using quantum mechanical calculations and molecular dynamics (MD) simulations [41]. The density functional theory (DFT) calculations elucidate the reaction mechanisms explaining CO_2_ uptake and cycloaddition. The cycloaddition reaction with carbon dioxide had a lower energy barrier when arginine was involved. Results from the MD simulations highlight the higher level of electrostatic interaction between [MOBMIM][Arg] and CO_2_, if compared to the other studied molecules. Moreover, the Lennard Jones interaction emerges as the dominant type of interaction in these systems. The diffusion coefficient for CO_2_ was highest when interacting with [MOBMIM][Gly].

Being imidazolium-based ionic liquids a popular family of these chemicals, it is not rare that a number of hydroxyl-functionalized imidazolium ionic liquids were prepared from the condensation of ethanolamine with glyoxal and formalin in the presence of acetic acid as a catalyst [42]. The chemical modification of the hydroxyl groups with epichlorohydrine added new hydroxylpropanoxychloride groups on the imidazolium cation, which formed a quaternary compound when N-methylimidazolium chloride was used to produce imidazolium acetate ionic liquids. The usefulness of the fabricated ionic liquids as single or mixed solvents with saline water to capture CO_2_ under atmospheric or 55.2 bar pressures was evaluated at room temperature. The data indicated that CO_2_ uptake depended on the ionic liquid structure (Table 3), with maximum uptake when pure DImIIL was used. The CO_2_ capture efficiency increased from 6.2 (15 min) to 16.8 (30 min) mol CO_2_/kg and from 9.1 (15 min) to 20.0 (30 min) mol CO_2_/kg at atmospheric and 55.2 bar pressures, respectively. In the presence of saline water (70/30), the maximum CO_2_ uptake decreases in the case of HIIL (5 mol/kg) and DImIIL (14.1 mol/kg) and increases in the case of CPIIL (8.0 mol/kg).

Utilizing monoethanol amine solutions, the catalytic effect of an imidazole series of ionic liquids ([BMIM][PF_6_], [BMIM][BF_4_], [BMIM][HSO_4_], [BMIM][NO_3_], [BMIM][CH_3_COOH], [BMIM][Cl], [BMIM] [Br], and [BMIM][I]) was evaluated with respect to the CO_2_ desorption rate, amount of gas released, and heat duty [43]. The BMIM][PF_6_] catalyst shows better catalytic activity for CO_2_ desorption (3.20 × 10^−4^ mol/L·s at 21.2 h) with respect to the use of solutions containing only MEA (2.92 × 10^−4^ mol/L·s after 22.3 h), reducing the relative heat duty by 17.59%. In addition, the catalytic applicability of [BMIM][PF_6_] for secondary (*N*-methyl monoethanolamine) and tertiary amine (2-(diethylamino) ethanol)) systems was evaluated, and the results were similar to those obtained with the primary amine. The reaction of the primary and secondary amines and CO_2_ is attributable to the zwitterion mechanism. As previously mentioned in this review, this is a two-step mechanism, including the zwitterion formation and carbamate breakdown. In the case of the tertiary amine, the base-catalyzed hydration mechanism is responsible for CO_2_ capture.

As in previous references, two imidazolium ionic liquids ([BMIM][PF_6_] and [BMIM][OAc]) are used for CO_2_ capture, in this case from biogas, in an absorption process assuming equilibrium at each stage [44]. After the capture, three processes are investigated, (i) solute-phase electroreduction, (ii) gas-phase electroreduction, and iii) solute-phase hydrogenation, in order to produce formic acid as the final product. The results showed that solute-phase electroreduction (USD 2103/ton) had the highest production cost, followed by gas-phase electroreduction (USD 986/ton) and CO_2_ hydrogenation (USD 868/ton). It was also shown that without any intervention, only hydrogenation can generate a profit. Gas-phase electroreduction results in the lowest CO_2_ emissions (0.7 kg CO_2_/kg HCOOH). In terms of production costs, and to reach a convenient parity with hydrogenation, a minimum current density of 417.3 mA/cm^2^ is recommended, though these costs can be reduced with an electricity cost reduction and carbon trading mechanism.

A combination of density functional theory–infrared (IR) conductor-like screening model for real solvents (COSMO-RS) and molecular dynamic (MD) methods was used to investigate the impact of hydroxyl functional groups on CO_2_ removal within dicationic ionic liquids (DILs) (C_5_(mim)_2_-(C_4_)_2_][NTf_2_]_2_, [C_5_(mim)_2_-(C_2_)_2_(OH)_2_][NTf_2_]_2_ [45]. The latter compound exhibited stronger ion–ion interactions, higher density, and reduced free volume, which resulted in a reduction in CO_2_ capture.

Tuning of azole-based ionic liquids for reversible CO_2_ capture from ambient air was investigated [46]. The synthesized ionic liquids were [N_2224_] with [Tiz], [Triz], [BeNIM] or [4-Br-Im] anions, [P_4446_] with [Im] or [BeNIM] anions and [P_4442_] with [Im] or [BeNIM] counter-anions. Through tuning the basicity of the anion as well as the type of cation, an ideal azole-based ionic liquid with both high CO_2_ capacity and good stability was synthesized. [N_2224_][BeNIM] exhibited the highest single-component isotherm uptake of 2.17 mmol/g at an atmospheric CO_2_ concentration of 0.4 mbar and 30 °C and in the presence of moisture.

A series of amino acid ionic liquids (AAILs) are fabricated via one-step hydrolysis of lactams, which may act as messengers to facilitate aqueous solutions of multi-tertiary amines’ fast and increasing CO_2_ capture [47]. The results indicated that [N_1111_][Maba]-PMDETA-50% (PMDETA= pentamethyldiethylenetriamine) showed a greater absorption rate constant value (0.282 min^−1^) than that of MDEA solution (0.043 min^−1^), demonstrating the activated role of AAILs. At 1.0 bar, [N_1111_][Maba]-PMDETA-50% showed an absorption capacity of 0.162 g/g, whereas at 0.1 bar, the uptake was 0.095 g/g; the above results were obtained at 40 °C.

Within the same type of chemical as in the previous reference, a series of protic amino acid ionic liquids (PAAILs) were synthesized through acid–base neutralization and an ion exchange reaction [48]. Among the synthesized PAAILs, [DBNH][Maba] (derived from 1,5-diazabicyclo [0,3,4]non-5-ene (DBN) and meta-aminobenzoic acid (Maba)) had the largest CO_2_ absorption capacity of 0.78 mol/mol (0.142 g/g) at 40 °C.

Sterically hindered amines (SHAs), such as 2-amino-2-methyl-1-propanol (AMP) and ionic liquids, are combining to form different sterically hindered amino acid ionic liquids (SHAAILs) [49]. Aqueous solutions of these compounds are investigated for CO_2_ capture. These absorbents presented remarkable physiochemical properties, CO_2_ upload of up to 0.92 mol/mol (40 °C and 1.0 bar), and a fast absorption rate (greater than AMP). Among them, [N_1111_][β-iPrNH-Ala] presented the best characteristics as a CO_2_ absorbent, which is attributable to its convenient steric hindrance and ionic nature. Analysis confirms that the SHAAILs undergo the carbamate-mediated rapid hydrolysis mechanism in absorbing CO_2_.

Two more works related to theoretical studies are outlined next. This reference investigates the efficacy of feedforward neural network and XGBoost models to screen ionic liquids, which can be used in CO_2_ capture [50]. Both models were integrated with either group contribution (GC), molecular structure descriptors (MSD), or hybrid GC-MSD to give performance comparisons. It was demonstrated that the XGBoost models performed better over feedforward neural network models, irrespective of descriptor types. Moreover, the XGBoost-GC-MSD model outperformed the artificial neural network with the group contribution (ANN-GC) and structure encoding multilayer perceptron (SE-MLP) models from the previous reference. The Shapley additive explanation analysis identified the top five influential input features: pressure, temperature, Chi2v, Chi0n, and BertzCT.

In this second work [51], three different machine learning (ML)-based models were investigated, i.e., gaussian process regression (GPR), LightGBM, and CatBoost, for predicting the solubility of CO_2_ in various ionic liquids. Three molecular descriptors, i.e., group contribution (GC), molecular structure descriptors (MSDs), and hybrid GC-MSD, were used in the three models. Overall, all models exhibited proficient CO_2_ solubility prediction in the ionic liquids, with performance varying based on descriptor type. The hybrid GC-MSD, and particularly the CatBoost-GC-MSD model, consistently outperformed others, attributed to GC-MSD incorporating a broader array of molecular feature information. As in the previous reference, the use of the Shapley additive explanation identified pressure, temperature, Chi0, Kappa2, and EState_VSA10 as the top five influential input features.

The mass transfer intensification and intrinsic mechanisms of different types and concentrations of metal salts (NaBF_4_, KBF_4_, KCl, and CuCl_2_) with task-specific ionic liquids (1-aminopropyl-3-methylimidazolium tetrafluoroborate ([APMIM][BF_4_]) aqueous solutions for CO_2_ removal process were investigated using a microchannel reactor [52]. The results showed that the presence of metal salts enhanced the interaction between metal cations and ionic liquid anions, thus decreasing the interaction between anions and cations within the ionic liquid molecules and promoting the chemical reaction between ionic liquid cations and CO_2_. From the above salts, the presence of KBF_4_ in the solution produced a greater effect for mass transfer in the CO_2_ absorption process. The mass transfer coefficient increases as the gas–liquid phase flow rate rises under slug flow conditions.

Three imidazolium ionic liquids, 1-butyl-3-methylimidazolium acetate ([BMIM][OAc]), 1-butyl-3-methylimidazolium 1,2,4-triazole ([BMIM][Tz]), and 1-butyl-2,3-dimethylimidazolium 1,2,4-triazole ([BMMIM][Tz]), were synthesized to absorb carbon dioxide by the formation of CO_2_ adducts [53]. These adducts can be in situ converted into dialkyl carbonates with alcohols (ROH, R = CH_3_, C_2_H_5_, and C_4_H_9_) in a diiodomethane medium under ambient temperature and pressure. The results indicate that [BMIM][Tz] exhibits the best CO_2_ absorption capacity and dimethyl carbonate (DMC) yield, where CO_2_ capacity is up to 0.22 g/g and DMC yield (based on methanol) is in the 20.2% order and 9.8% based on CO_2_. The structures of the imidazolium ionic liquids have significant effects on the absorption of CO_2_ and subsequent transformation processes, because these structures influence the binding sites for CO_2_ absorption, which can combine with the gas to form CO_2_ adducts, zwitterion [BMIM-CO_2_], and anionic [Tz-CO_2_] species. Further analysis shows the structural effects (binding sites and steric hindrance) of the ionic liquids on the activation of CO_2_ and methanol, through the elongated C[dbnd]O bond lengths of CO_2_ adducts and O-H bond lengths of methanol in ionic liquid–methanol complexes, respectively, leading to different DMC yields.

The energy analysis and economic evaluation of CO_2_ capture onto ionic liquids on the basis of thermodynamic models are of importance, and this is why they were considered in the next reference [54]. The process simulation resulted in a chemical absorption-dominated hybrid solvent, consisting of functional ionic liquid choline triazole ([Cho][Triz]) and sulfolane (TMS) to separate CO_2_ from shale gas. The total gas capture energy consumption of 1.65 GJ/t CO_2_ and the cost of 48.07 USD/t CO_2_ were achieved using the absorbent when the mass fraction of the ionic liquid was 60 wt%. Compared with the commercial 30 wt% MDEA carbon capture process, it reduced the energy consumption and economic cost by 64.16% and 45.59%, respectively.

In this reference [55], the steric effects of amino acid ionic liquid anions (based on glycine, alanine and valine) with various chemical structures for CO_2_ capture were investigated. It was shown that carbamate formation was attributable to a two-step reaction pathway with a zwitterion intermediate undergoing dynamic proton transfer. It can be concluded that the favored structural flexibility due to the reduced steric hindrance of the zwitterion leads to enhanced intermolecular interactions, facilitating proton transfer to nearby amino acid ionic liquid anions for carbamate formation. Carbamate formation is kinetically favored, due to a lower barrier height, as the degree of steric hindrance of anions decreases in the order valine > alanine > glycine.

Four protic ionic liquids were prepared from superbase 1,8-diazabicyclo [5.4.0]undec-7-ene (DBU) and 1,5-diazabicyclo [4.3.0]-5-nonene (DBN) as cations, with imidazole (Im) and pyrazole (Pyr) as anions [56]. The results showed that CO_2_ primarily interacted with the anions of the protic ionic liquids through van der Waals forces, while the cations and anions of these chemicals mainly engaged in strong hydrogen-bonding interactions. In addition, the anions primarily serve as the absorption reaction sites for CO_2_, with their molecular centers of mass being the closest. Molecular density simulations have also shown that CO_2_ molecules are preferentially accumulating at the gas/protic ionic liquid interfaces and that chemisorption governs the gas capture at low pressures in these types of ionic liquids. Among the studied systems, [DBUH][Pyr] was the most reacting system with CO_2_, while [DBUH][Pyr] showed lower regeneration energy.

In this reference [57], a series of density functional theory (DFT) calculations and molecular dynamics simulations were conducted to investigate the role of ethyl-3-methylimidazolium acetate ([EMIM][OAc]) in CO_2_ capture by poly(ethylenimine (PEI). The results showed that the formation of hydrogen bonds between the ionic liquid anion and the amino groups of PEI primarily governed the addition of the ionic liquid to PEI. During the CO_2_ absorption process, the ionic liquid anion absorbed CO_2_ but also produced a dehydrogenation effect on the amino group of PEI, facilitating the interaction between PEI and CO_2_. It was shown that, in the presence of the ionic liquid, the viscosity of PEI was reduced, and the diffusion of CO_2_ increased due to the increase in the absorption rate. CO_2_ capture by the mixture ionic liquid + PEI depended on the temperature, decreasing CO_2_ uptake as the temperature increased from 25 °C to 60 °C (Table 4).

Three anion-functionalized proton ionic liquids were prepared from 1,5-diazabicyclic [4.3.0]non-5-nonene (DBN) and 2-hydroxypyridine (2-OP), 3-hydroxypyridine (3-OP) or 4-hydroxypyridine (4-OP) [58]. The results showed that [DBNH][2-OP] with an oxygen atom located in the ortho position of the nitrogen atom presented lower viscosity and larger free volumes as well as stronger alkalinity, which resulted in a CO_2_ capture capacity of about 1.4 mol/mol ionic liquid (about 0.3 g CO_2_/g) at 30 °C.

Triazole anion-functionalized ionic liquids (TAFILs) were formulated by combining low-molecular-weight cations [Cho] or [N_222_] with triazole anions containing a nitrogen-electronegative site and further mixing with solvents to form physicochemical absorbents [59]. The results indicated that [Cho][Triz]/TMS (sulfolane) (80 wt%/20 wt%) upload 0.125 g CO_2_/g absorbent, which is equivalent to that presented by a 30 wt% MEA solution at 40 °C and 1 bar but with an enthalpy value (−35.76 kJ/mol) less than half that of 30 wt% MEA solution. CO_2_/CH_4_ selectivity (191.0) was obtained for the [Cho][Triz]/TMS absorbents. The results were explained in terms of the relatively weak chemical and physical interaction between CO_2_ and TAFIL absorbents.

### 2.2. Ionic Liquids in Biphasic Systems

The combination of two aqueous solutions of polymer/polymer, polymer/salt or salt/salt results in the formation of aqueous biphasic solvents [60]; moreover, about 20 years ago, ionic liquids were introduced as a replacement for the polymer in these solvents [61]. In the CO_2_ capture process, the gas (or the product) accumulated in one phase (rich phase).

An ionic liquid non-aqueous biphasic absorbent ([TEPAH][CPL]/EG/DGDE)) composed of tetraethylenepentamine (TEPA) caprolactam (CPL), ethylene glycol (EG), and diethylene glycol dimethyl ether (DGDE) was proposed for CO_2_ capture [62]. The maximum gas uptake of the optimal absorbent reached 1.88 mol/mol, with 40% of the saturated phase volume containing 98% of the mass percentage of CO_2_ products. The regeneration energy consumption was 1.88 GJ/t CO_2_, approximately 51% lower than that of 30 wt% monoethanolamine (MEA) aqueous solution. The amino group on the cation [TEPAH] reacted with CO_2_ to form carbamate, and the anion [CPL] assisted the proton transfer during the reaction. The phase separation behavior of the absorbent was governed by the polarity difference between the CO_2_ products and the solvent.

A dual-functionalized ionic liquid ([DETA][Lys]) was synthesized using neutralizing routes from diethylenetriamine (DETA) and lysine (Lys) [63]. It could remove CO_2_ through a two-stage reaction, achieving a loading capacity of up to 2.16 mol/mol. N-methyl-2-pyrrolidone (NMP) was demonstrated to be a suitable phase splitting agent for [DETA][Lys]-based biphasic solvent by tailoring the polarity and hydrogen bonding effect. Thus, DETA-carbamate, Lys-carbamate, and HCO_3_^−^/CO_3_^2−^ during CO_2_ absorption were formed. These carbamate derivatives accumulated in the lower layer, while most of the NMP concentrated in the upper layer. The CO_2_-rich phase took up 53.84% of the total volume and 99.03% of the captured gas molecule, with a gas uptake of 4.78 mol/L.

Also, the next two references used dual-functionalized ionic liquids such as T-Im and D-Im. These were synthesized and used as promoters in low-reactive aqueous methyldiethanolamine to fabricate a CO_2_-capturing blend system [64,65]. The gas absorption kinetic performance of these blends was investigated in the 30−55 °C temperature range and different pressures (210−710 kPa). The results indicated an increase in the initial absorption rate with the increase in the DFIL concentration (2.5–10 wt%). It was assessed that carbamate formation increased with the increase in T-Im concentration in the blend, whereas T-Im was more favorable towards CO_2_ capture than D-Im for experiments carried out at high temperatures. Modeling of the rate kinetic using the zwitterion and base-catalyzed hydration mechanisms was performed.

In this reference [66], simulation was carried out to model the gas–liquid equilibrium of CO_2_ in two aqueous mixtures of ionic liquids, 1-butyl-3-methyl imidazolium acetate ([BMIM][Ac]) and amines: [BMIM][Ac] + 1-(2-aminoethyl) piperazine (AEP) and [BMIM][Ac] + bis (3-aminopropyl) amine (APA). It was concluded that the aqueous mixture (2.0 m [BMIM][Ac] +1.0 m APA) exhibited the highest CO_2_ removal, followed by (2.0 m [BMIM][Ac] +1.0 m AEP) compared to the results derived from the use of all the investigated solvent mixtures.

This reference investigated the use of an aqueous-based biphasic solvent, comprising methyl monoethanolamine (MMEA), N-methyldiethanolamine (MDEA), and 1-butyl-3-methylimidazolium tetrafluoroborate ([BMIM][BF_4_]), on CO_2_ capture from a mixture of CO_2_ and CH_4_ [67]. In this investigation, the ionic liquid was used as a phase separator. This phase separation is based on the incompatibility of the ionic liquid, with the carbamates produced as a consequence of the CO_2_ capture by the amine mixture. The experimental results showed that the impact of inlet flow rate, stirring rate, and initial methane concentration on biogas upgrading was negligible. The utilization of a mass ratio of 3:1 for MMEA:MDEA produced the best results in terms of methane purity (>96%), CO_2_ saturated uptake (0.62 mol CO_2_/mol amine) (Table 5), low CO_2_ heat of desorption (61.45 kJ/mol CO_2_), best CO_2_/CH_4_ selectivity, and economic efficiency. In the original work (Section 3.6.), the usefulness of the ionic liquid was mentioned; no further data about its utilization, i.e., concentration, were mentioned in the published manuscript.

The ionic liquid (diethylenetriamine-3-hydroxypyridine [DETA][3HPyr]) presenting multiple active sites was combined with diethylene glycol monobutyl ether (DGME) and water to prepare a biphasic solvent and used for CO_2_ absorption [68]. The results showed that after the absorption process, the solvent changed from a single to a two-phase system, with the presence of highly self-concentrated absorption products in the rich phase. The volume of the rich phase was about 36.3% of the total solvent volume, whereas its viscosity decreased to 28.1 mPa·s, with an absorption capacity of 2.67 mol/kg. The stable presence of carbamic acid in the system was confirmed, thus promoting the absorption capacity. Density functional theory calculations revealed that DGME stabilized carbamic acid through intermolecular hydrogen bonding interactions, and highly polar ions generated during absorption and uneven charge distribution between ions dominated the phase-change process and increased the water content in the rich phase.

Dual-functionalized ionic liquid [DMAPA][TZ] was synthesized as a primary absorbent and mixed with phase separation accelerators such as poly(ethylene glycol) dimethyl ether (NHD) or propylene carbonate (PC) as biphasic solvents to achieve high efficiency, energy savings, and corrosion inhibition of post-combustion CO_2_ capture [69]. The results demonstrated a transition from a homogeneous solvent to a biphasic system upon gas absorption, where the majority of CO_2_ was concentrated in the rich phase, attributed to the formation of hydrogen bonds. Interaction between the ionic liquid and PC is significantly stronger than that with NHD, resulting in a reduced absorption capacity of IL-PC in comparison to IL-NHD, while NHD effectively suppressed the formation of HCO_3_^−^/CO_3_^2−^. The utilization of the ionic liquids inhibited corrosion through the adsorption–passivation mechanism; the corrosion rate of the CO_2_-rich phase in IL-NHD relative to 20# carbon steel was 0.1896 mpy, which is 1/865 of the rate of the saturated 5 M methyl ethanol amine (MEA) solution.

A biphasic solvent based on diethylenetriamine serine ionic liquid/polyethylene glycol dimethyl ether/water ([DETA][SER]/NHD/H_2_O) was developed [70]. The viscosity of the ionic liquid was regulated by weakening the hydrogen bonding and van der Waals interactions within the pure ionic liquid by adding NHD and H_2_O to [DETA][SER]. The optimal mass ratio of [DETA][SER]/NHD/H_2_O was determined to be 20/40/40 wt%. The viscosity of this solvent was found to be 7.82 mPa·s, with a total absorption uptake of 1.26 mol/mol of ionic liquid, and where the rich-phase load accounts for 99% of the total load and occupies 37% of the volume. CO_2_ was captured by the formation of zwitterions from the reaction of the primary amines on the cationic [DETA] and anionic [SER] species with CO_2_. Subsequently, proton transfer and hydrolysis of the carbamate esters were observed. NHD was found to promote phase separation without participating in the various chemical reactions.

A strategy for biphasic solvent preparation was investigated by combining amine blends of 3,3′-diaminodipropylamine (DADPA) or N-methyldiethanolamine (MDEA) and composite agents, such as 1-butyl-3-methylimidazolium bis(trifluoromethylsulfonyl)imide ([BMIM][NTf_2_) and sulfolane (TMS) [71]. The ratio of DADPA:MDEA:TMS:BN:H_2_O (DMTB) used was 2:6:2:1:2. Experiments showed that the DMTB solvent exhibited a high CO_2_ capacity (4.59 mol/L in the 50 vol% rich phase) and an efficient phase splitting rate (within 3 min). The results indicated the formation of anionic DADPACOO species in the CO_2_-saturated phase in the presence of water rather than bicarbonate, while observing dissociative behaviour in MDEA and TMS. Therefore, the phase separation analysis revealed that the products of protonated amines and carbamic acid species were highly polar and preferred to dissolve in the water phase.

In order to gain a better understanding about bicarbonate formation and its influence on phase separation in the water-[N_1111_][Gly]/EtOH system, further investigations were carried out through quantum chemical calculations employing density functional theory, with reaction rate constants determined via transition state theory [72]. Among the various reaction pathways, the base-catalyzed CO_2_ hydration reaction becomes predominant, i.e., one-step reaction involving anionic [Gly] species, H_2_O, and CO_2_. A hydrogen atom in water was transferred to the amino group of glycine anion, forming protonated [Gly]^−^ and hydroxide combined with CO_2_, generating bicarbonate. Furthermore, when the water content was greater than 5 wt%, an increase in water content promoted the reduction in hydrogen bonding among products and enhanced the hydrogen bonding of the solvent, decreasing product self-aggregation.

Despite the fact that biphasic absorbents composed of tetraethylenepentamine, 1-ethylimidazole, and H_2_O (TEH) exhibited remarkable absorption rates and CO_2_ uploads, some problems arise in relation to precipitation in the enriched phase, attributable to strong interactions among absorption products. To avoid this issue, six amino acid ionic liquids (AAILs) were introduced as split-phase regulators in TEH, developing a liquid–liquid biphasic absorption system, TEH-AAILs [73]: alanine [Ala], glycine [Gly], serine [Ser], γ-aminobutyric acid [Gaba], proline [Pro], α-aminoisobutyric acid [Aib]. The formulation of TEH-[Ser] and TEH-[Ala] presented the best performance in terms of CO_2_ uptake (about 1.8 mol/kg after 40 min, 101 kPa and 40 °C), though the former presented the best loading rates at shorter times (up to 10–15 min). Bridged hydrogen bonding structures between [Ser]–CO_2_ and H_2_O were responsible for this efficiency. Desorption was carried out below 2.5 kPa and 85 °C, with a regeneration efficiency exceeding 85%.

An amino acid-based ionic liquid ([N_1111_][Ala]) (Ala: alanine) was used as a trigger to start the phase separation of 2-(2-aminoethylamino) ethanol (AEEA) and 1-ethylimidazole (Eim) solutions (AEH) and to favor CO_2_ capture [74]. Due to the accompanying phase change, the CO_2_ load of AEEA-Eim-H_2_O-[N_1111_][Ala] (AEHI) is 1.47-times higher than that of AEH. The water-shell layer around [Ala]–CO_2_ and hydrogen bonding were the key reasons for the phase separation. The formation of the water-shell layer expelled Eim from the rich phase and, thus, facilitated the proton transfer process, which not only favored the phase separation process but also decreased the regeneration energy consumption.

An ionic liquid derived from arginine, triethylmethylammonium argininate ([N_2221_][Arg]), was synthesized and used to capture CO_2_ in pure form and from the open air [75]. In order to improve some drawbacks derived from the use of these AAILs (high viscosity and low mass transfer), this ionic liquid was mixed with a binary mixture of water and dimethyl sulfoxide (DMSO), with a variable volume ratio. Increasing the volume of DMSO in water, the CO_2_ uptake gradually decreased, reaching a minimum in pure DMSO and the maximum in an aqueous solution (Table 6). This [N_2221_][Arg] ionic liquid in the aqueous medium was used to remove CO_2_ from direct air. The open-air CO_2_ uptake concentration value was near 1.01 mol/mol in the aqueous solution of arginine-based ionic liquid.

The efficiency of CO_2_ absorption was investigated in aqueous MDEA solutions, and the energy consumption of the process was reduced by designing combined absorbent systems [76]. For this, an ionic liquid based on the amino acid glycine, known as bis(2-hydroxyethyl)dimethylammonium glycinate ([M_2_E_2_A][Gly]), was synthesized and investigated as a carbon dioxide absorbent. The absorption capacity of [M_2_E_2_A][Gly], its aqueous solutions and amine + ionic liquid aqueous mixtures was evaluated gravimetrically at different temperatures and concentrations of components. The results indicated that there was an increase in both the absorption capacity and the rate of chemical reaction with carbon dioxide when using this ionic liquid as an additive. The H_2_O/MDEA/[M_2_E_2_A][Gly] formulation (50/30/20 wt%) exhibited the highest absorption capacity of 2.98 mol CO_2_/kg solution.

Two triazole-functionalized ionic liquids ([N_2222_][1,2,4-Triz] and [N_2222_][1,2,3-Triz]) were used and combined with imidazole organic solvents as phase separation agents to form a series of systems aimed at CO_2_ capture [77]. Within a 5:4 mass ratio formulation ([N_2222_][1,2,4-Triz]:1-methylimidazole (N-MI)), CO_2_ loading is 0.112 g/g of solvent at 40 °C and 1 bar, in which the CO_2_-rich phase accounts for 95% of the total CO_2_ capacity and 60% of the total volume. The results indicated that [N_2222_][1,2,4-Triz] can react with CO_2_ to produce carbamate that existed in the CO_2_-rich phase, and, as a consequence of the gas absorption, the original homogeneous liquid evolved into a liquid–solid phase due to the difference in polarity between imidazole organic solvents and carbamate products and also to strong intermolecular hydrogen bonding between carbamate products.

### 2.3. Remarks About Absorption and Ionic Liquids

Though it is difficult to compare results due to the different experimental conditions used in the various investigations, Table 7 (monophasic systems) and Table 8 (biphasic systems) summarize a number of CO_2_ uptake results in order to provide knowledge about the performance of different systems used in CO_2_ capture.

## 3. Adsorption and Ionic Liquids

When this technology is used for CO_2_ capture, the ionic liquid is immobilized onto the solid material or part of the material intimate structure. The active part for CO_2_ capture is not only the ionic liquid, because, very often, the characteristics of the solid material influence this capture.

Agave bagasse fiber-derived biochar (BCw) was impregnated with 1-butyl-3-methylimidazolium acetate ionic liquid. This adsorbent presented the next CO_2_ removal capacities at atmospheric (1.47 mmol/g at 25 °C and 1 bar) and moderately high pressure (1.32 mmol/g at 25 °C and 8.5 bar) under dynamic and static systems, respectively [78]. Biochar-IL composites improved the CO_2_ capture capacity, kinetics, and selectivity (CO_2_/N_2_) by 4–90% compared with the bulk adsorbents. By increasing the pressure from 1 to 8.5 bar in a static high-pressure system, the CO_2_ capture capacity and kinetics of impregnated-biochar improved two times. Additionally, the biochar-IL composites were regenerated under a pressure swing adsorption (PSA) arrangement, where more than 70% of the desorption was attributed to the depressurization of the system. Gas–solid physisorption and gas–liquid chemisorption at low pressure were responsible for the CO_2_ loading onto the adsorbent. Apparently, CO_2_ capture was not only attributed to the textural properties of the biochar but also to its surface chemistry.

This reference was related to carbon capture and storage (CCS), in which the CO_2_ adsorption capacities of a series of elastomer poly(ionic liquid) (PILs) with imidazolium cations having different alkyl chains were investigated [79]. Three PIL formulations were investigated: PIL-Me (methyl), PIL-nBum (n-butyl), and PIL-nHex (n-hexyl). The best results were obtained with PIL having methyl chains (Table 9).

To overcome some negative issues (low porosity, limited batch conversion capacity, and difficulties in reuse) relative to the utilization of poly(ionic liquids) on CO_2_ capture, AEROPIL catalysts obtained from the integration of poly(ionic liquid)s in chitosan-based aerogels were proposed to remediate the above issues [80]. Working in batch mode, AEROPILs show moderate yields for CO_2_ conversion; however, high catalytic activity was achieved when AEROPILs were used to catalyze the CO_2_ cycloaddition reaction to epoxides in packed-bed reactors operating under continuous flow.

The effect of encapsulating various imidazolium ILs in porous ZIF-8 was reported to investigate the adsorption mechanism of CO_2_ onto the composite adsorbents [81]. Thus, a number of anions, including bis(trifluoromethylsulfonyl)imide [NTf_2_], methanesulfonate [MeSO_3_], and acetate [AC], were combined with a 1-ethyl-3-methylimidazolium [EMIM] cation. These [EMIM]-based ILs@ZIF-8 composites were computationally investigated to identify suitable materials for CO_2_ capture. The results demonstrated that the incorporation of ILs strongly affects the adsorption capability of CO_2_, which is highly dependent on the nature of the ILs inside the ZIF-8 framework, and demonstrated that the incorporation of ILs into ZIF-8 led to better CO_2_ uptakes if compared to isolated ILs and pristine ZIF-8. The [EMIM][NTf_2_]@ZIF-8 composite exhibited higher activity and stability toward the adsorption of CO_2_ than the other composites, which was attributed to significant charge transfer and strong interactions between the fluorinated IL and ZIF-8.

This investigation was performed in order to asses CO_2_ adsorption capacity and the CO_2_/N_2_ selectivity of different di-cationic ionic liquids (based on dichlorate 1,8-diimadozole-3,6-dioxaoctane-bis-(propyl)triethoxysilane (DIL_2Cl)) immobilized in commercial mesoporous silica support (SBA-15) [82]. The highest CO_2_ adsorption capacity and CO_2_/N_2_ selectivity were obtained for sample SBA@DIL_2FeCl_4_ (at 1 bar and 25 °C, 57.31 (±0.02) mg CO_2_/g, 12.27 (±0.72) mg CO_2_/g). Though the results demonstrated similar adsorption capacity between the pristine material and the IL-bearing adsorbents, the presence of the ionic liquids improved the CO_2_/N_2_ selectivity by approximately 3.8-times. Compared to the results obtained with monocationic ionic liquids immobilized on mesoporous supports and the same percentage of ionic liquid content, these results show that the use of dicationic-ionic liquids leads to improved CO_2_ selectivity due to the increased availability of coordination sites.

Porous poly(ionic liquid)s was formed by the incorporation of dual-hydrogen-bond donors (amide and hydroxyl groups) and nucleophiles (Br^−^) as multiple effective active sites for the cycloaddition reaction between CO_2_ and epoxides [83]. The presence of dual-hydrogen-bond donors provides two active sites for synergistic catalysis with epoxides and also increases CO_2_ adsorption capacity. By regulating the ratio between the ionic liquid monomer and divinylbenzene, a hierarchical porous structure favorable for the reaction was fabricated, resulting in optimized catalytic activity comparable to that of bulk ionic liquid monomers. Furthermore, this catalyst exhibits structural stability exhibited after five consecutive use adsorption cycles. Analysis of the material indicated that the –OH group of the ionic liquid, C–O group of amides, and –CH_2_ produced during polymerization remained unaltered. Moreover, the adsorbents retained their irregular highly dense granular morphology after the recycling tests. It was concluded that in the catalytic mechanism, the dual-hydrogen-bond donors and bromide anions synergistically promoted ring-opening reactions of epoxides; at the same time, amide groups served as basic sites to favor CO_2_ uptake during the reaction process.

The formation of composites between metal–organic frameworks and ionic liquids suggested the creation of new materials, amenable for use in the removal of CO_2_ (and other toxic gases). On this basis, a core–shell composite utilizing ionic liquids and metal–organic frameworks based on the dissolution–diffusion mechanism was fabricated [84]. The affinity of ionic liquids towards various gas molecules, particularly their strong compatibility for CO_2_, allow only this gas to be loaded, while rejecting other gases. CO_2_ exhibits strong interactions with the amino groups present in [TETA][Lys] (formed by reaction of triethylenetetiamine and L-lysine). The composite presented a good separation selectivity for CO_2_/C_2_H_2_, CO_2_/CH_4_ and CO_2_/N_2_. Breakthrough experiments confirmed the effectiveness of [TETA][Lys]@ZIF-8 in removing trace amounts of CO_2_ from various gas mixtures (Table 10).

Acidic and basic sites of catalysts play a key role in CO_2_ capture and activation. In this reference [85], Zr, N-ZnO/ZnAl-LDH-IL composites in ionic liquid (tetraethyl ammonium-based) and methanol systems were formulated and utilized to catalyze, from ethylene glycol (EG) and CO_2_, the synthesis of ethylene carbonate (EC). The composites showed stronger basic sites due to the presence of reactive groups on the catalyst surface through Zr doping, resulting in an increase in pyridinic-N groups. Carbon atoms near to pyridinic-N, as strong basic sites, activated CO_2_ and EG. The replacement of Zr in the catalyst by La, Fe, Ce or Cu did not increase EC evolution but decreased it, with the best results derived from the use of Zr, followed by La = Ce > Fe > Cu.

Another example of the joint use of metal–organic frameworks and ionic liquid is given. Composites for carbon dioxide separation consisting of two task-specific ionic liquids, namely, tetramethylgunidinium imidazole [TMGH][IM] and tetramethylgunidinium phenol [TMGH][PhO], impregnated in ZIF-8 were developed and compared with results derived from the use of pristine ZIF-8 [86]. Though pristine ZIF-8 presented better CO_2_ uptake concentrations (especially in the 0.4–1 bar pressure range) than the two composites, the formulation [TMGH][IM]@ZIF-8 demonstrated better CO_2_ adsorption performance if compared with [TMGH][PhO]@ZIF-8. This is attributed to its strong attraction toward CO_2_, resulting in a higher CO_2_/CH_4_ selectivity of 110 (0.01 bar), a value which decreased to 12 with the increase in the pressure (1 bar); in any case, these are better than the values presented by the pristine framework. The dual-site Langmuir (DSL) model was developed to fit experimental adsorption data:

(3) $$q_{e}=\frac{n_{A}b_{A}P}{1+b_{A}P}+\frac{n_{B}b_{B}P}{1+b_{B}P}$$

where q_e_ (mmol/g) is the equilibrium uptake of the gas, P (bar) is the equilibrium pressure and n_A_, b_A_, n_B_ and b_B_ are the fitting parameters. In the present case, the values for CO_2_ are n_A_ 0.6118, b_A_ 0.4604, n_B_ 100 and b_b_ 0.000302 with r^2^ 0.9998. The respective values for CH_4_ are 1.0, 0.0068, 1, 0.6883 and 0.9998.

The design and synthesis of improved mesoporous silica-supported ionic liquid-based adsorbents for carbon dioxide capture are of interest. Thus, 1-ethyl-3-methylimidazolium tetrafluoroborate, 1-ethyl-3-methylimidazolium ethyl sulfate and 1-ethyl-3-methylimidazolium methylsulfate ionic liquids were supported on the surface of mesoporous silica to study its performance in this capture [87]. Theoretical results show the strong interaction of molecular bodies with CO_2_ gas molecules, which is evident from the experimental findings. The results demonstrate that the increase in CO_2_ uptake is attributed to weaker cation/anion pair interactions, favoring lattice expansion and greater CO_2_ interstitial spaces.

The role of defective UiO-66-NH_2_ and its ionic liquid-encapsulated counterparts were investigated by using hydrochloric acid as a defect modulator, followed by post-synthetic ionic liquid encapsulation for CO_2_ capture [88]. The defective UiO-66-NH_2_ showed an enhanced carbon dioxide uptake capacity compared to its non-defective counterpart because of the improved microporous structure. It was found that there exists an optimum linker deficiency, yielding the highest CO_2_ capture performance due to enhanced textural properties, overcoming the negative effect of reduced CO_2_-philic amine groups. Furthermore, ionic liquid-encapsulated UiO-66-NH_2_ showed a slight improvement in CO_2_/N_2_ selectivity but exhibited a notable reduction in CO_2_ uptake capacity because of decreased textural properties. In the investigation, 1-methylimidazole [1-MIM] and 1-bromobutane were used as precursors to form the ionic liquid [BMIM][Br].

Another composite was formed by incorporating magnesium and piperazinium-based solid ionic liquids (SoILs) into HKUST-1 to favor its water stability and selective CO_2_ capture performance [89]. An eco-friendly process was used in the synthesis of bimetallic Mg(x)-HKUST-1; the process used water as the unique solvent with stirring and microwave treatment. The SoILs were incorporated into the pore structure of Mg(15)-HKUST-1 through wet impregnation. Different ratios of Mg were used for the synthesis. Among them, 15% Mg exhibited the highest improvement in CO_2_ capacity (5.32 mmol/g) and CO_2_/N_2_ selectivity (27) compared to HKUST-1 (CO_2_ capacity 4.2 mmol/g; CO_2_/N_2_ selectivity 17). Once Mg(15)-HKUST-1 was impregnated with 10% SoIL, the CO_2_ capture performance was further improved. Moreover, 10% SoIL/Mg(15)-HKUST-1 and Mg(15)-HKUST-1 maintained, respectively, 92.4% and 85.3% CO_2_ adsorption capacity after 20 days of exposure to a humid environment (60% RH) at 25 °C, improving the 26% shown by HKUST-1.

Ionic liquid tetraethylammonium glycine ([N_2222_][Gly]) and a porous molecular sieve (SBA-15) were combined to fabricate a porous amino acid ionic liquid composite 60 wt% [N_2222_][Gly]@SBA-15 with microporous and ultra-microporous structures and used to investigate the dynamic (fixed-bed column) CO_2_ adsorption breakthrough in various conditions [90]. The results indicated that the CO_2_ adsorption capacity was up to 1.8 mmol CO_2_/g at 25 °C, 1 bar and zero humidity. This loading capacity increases to 2.4 mmol CO_2_/g at 15% RH due to the mechanism change of the CO_2_ reaction with primary amine from 2:1 under dry gas to 1: 1 stoichiometry in the presence of moisture (Table 11).

Further, CO_2_ adsorption breakthrough behaviors were fitted to the Avrami kinetic model:

(4) $$q_{t}=q_{e}[1-e^{-(k_{A}t{)}^{n_{A}}}]$$

where q_t_ is the CO_2_ uptake at an elapsed time, q_e_ is the gas molecule uptake at the equilibrium (saturation) time, whereas k_A_ and n_A_ are the adsorption rate and constant of the model, respectively. The CO_2_ adsorption rate of 60 wt% [N_2222_][Gly]@SBA-15 was controlled by intra-particle diffusion. The temperature for CO_2_ desorption was 90 °C.

Of the four types of porous liquids (I, II, II, and IV) [91,92,93,94], porous organic cage (POC)-based type III porous liquids, and especially those PCOs solvated in ionic liquids, were used less in terms of CO_2_ capture. Thus, this reference investigated the CO_2_ capacity of porous liquids composed of the POC cluster and [BPy][NTf_2_] (N-butylpyridinium bis((trifluoromethyl)sulfonyl)imide) or [BMIM][NTf_2_] (1-butyl-3-methylimidazolium bis(trifluoromethyl)sulfonyl)imide) [95]. The results indicated that the CO_2_ capacity of porous liquids containing [BPy][NTf_2_] was higher than that of the latter POC-ionic liquid formulation. Dynamical and structural analyses indicated that the CO_2_ diffusion rate was lowered by the interface between the solvent and the POC cluster in both systems. In the case of the imidazolium derivative, the lower CO_2_ capture was attributable to the ring part of cation molecules being concentrated above the cage window, hindering the CO_2_ entering the POC cluster inside.

A series of hydroxyl-functionalized ionic hyper-crosslinked polymers were prepared through a one-step Friedel–Crafts reaction, involving hypoxanthine (HX) and benzimidazole (BI) as the monomers, along with various halohydrocarbon crosslinking agents [96]. These polymers presented a high specific surface area (558–1480 m^2^/g), microporous structure, and unique ion sites. Their CO_2_ adsorption capacity of 157.5 mg/g at near 0 °C and 1 bar is of interest. Additionally, these polymers function as recyclable catalysts in the cycloaddition reaction of CO_2_ and epoxides, enabling the conversion of CO_2_ into cyclic carbonates, with rates up to 99%, even without a co-catalyst. A mechanism investigation revealed that the introduction of hydroxyl groups in the polymer is the key to improving catalytic activity through a synergistic catalytic effect.

The functionalization of ordered mesoporous silica MCM-48 with two amino acid-based ionic liquids (AAILs) ([Emim][Gly] and [Emim][Ala]) produced a couple of composites (AAILs@MCM-48), which were used to investigate their performance on the CO_2_ capture and CO_2_/N_2_ separation [97]. Under post-combustion flue gas conditions, both composites improved CO_2_ capture in comparison to pristine MCM-48. Maximum CO_2_ uptake was yielded with the 40 wt.%-[Emim][Gly] composite, with values of 0.74 and 0.82 mmol/g at 0.1 and 0.2 bar, respectively, and 30 °C. The CO_2_/N_2_ selectivity of the composites was also improved at 0.1 bar, with a value of 17 for the above composite formulation, which compares very well to the value of 2 of the unmodified MCM-48. [Emim][Gly] and [Emim][Ala] corresponded to 1-ethyl-3-methylimidazolium glycine and 1-ethyl-3-methylimidazolium alanine formulations, respectively.

The problem associated with the viscosity is always present when ionic liquids are used, in any field. Trying to mitigate this problem, ethylene glycol, propylene glycol, 1,3-propanediol, and diethylene glycol were added to three different ionic liquids: 1-ethyl-3-methylimidazolium 2-cyanopyrrolide ([EMIM][2-CNpyr]), 1-ethyl-3-methylimidazolium tetrafluoroborate ([EMIM][BF_4_]), and 1-butyl-3-methylimidazolium tetrafluoroborate ([BMIM][BF_4_]). This was to decrease (averaging 51%) the viscosity of pristine ionic liquids [98]. Since the encapsulated mixtures [EMIM][2-CNpyr]/1,3-propanediol and [EMIM][2-CNpyr]/diethylene glycol presented the lowest volatility (0.0019 and 0.0002 mmol/h at 25 °C, respectively), they were further investigated for CO_2_ capture. These results showed that [EMIM][2-CNpyr]/diethylene glycol capsules had higher uptake capacity at initial carbon dioxide concentrations of near 500 ppm (0.66 against 0.47 mol of CO_2_/kg when 1,3-propanediol was used). These results indicated that glycols can be effective not only to reduce ionic liquid viscosity while increasing physisorption sites for CO_2_ removal but also that encapsulation was useful to inhibit the evaporation of volatile modifiers.

Guanidine-functionalized basic binuclear poly(ionic liquid)s (P-DBTMGH) were prepared via free radical copolymerization [99]. The introduction of guanidine ionic liquids into the framework of GB-PILs improved the alkalinity in the structure, thus favoring CO_2_ surface adsorption and activation ability. Bromide-containing binuclear sites help to increase the ion density of bromide and promote the occurrence of ring opening. Using epichlorohydrin as a probe substrate, P-DBTMGH could efficiently catalyze the cycloaddition reaction of the simulated flue gas (15 vol% CO_2_ + 85 vol% N_2_), without any co-catalyst or solvent, and chloropropene carbonate (CPC) with a selectivity and yield of 99% and 96% was, respectively, obtained. Theoretical interpretations explained the synergistic effect of guanidine basic sites and bromide-containing binuclear sites on the CO_2_ activation and ring opening of epoxides, which provide a choice for high-value utilization of low-partial-pressure CO_2_ from flue gas.

The ionic liquid (1-carboxypropyl-3-methylimidazolium bromine (1-C-3-M)) was grafted onto a metal–organic framework without any additives and in a single-step operation [100]. In this composite, an amine bond through reaction of the carboxyl group in the ionic liquid with the amino group in the framework was formed. Thus, a composite material, MIL-101-NH_2_-1-carboxypropyl-3-methylimidazolium bromine (1-C-3-M@MIL-101-NH_2_), was the result. This composite, employed as a catalyst for CO_2_ cycloaddition, reached a target product PC (propylene carbonate) yield of 91.7% under the conditions of 80 °C, 1.0 MPa, and a reaction time of 4 h and without the addition of any co-catalysts or solvents. These results improved the respective one derived from the utilization of the pristine framework (25.6%). The catalytic activity benefitted from the synergistic effect of Lewis acidic Cr centers and nucleophilic bromide anions, as well as the high specific surface area and CO_2_ affinity of 1-C-3-M@MIL-101-NH_2_.

New composites, such as ZIF-8 structures with two functionalized ionic liquids (1-propyl-3-methylimidazole hydrochloride ([C_1_MIm]Cl), and tetraethylenepentamine-2-methylimidazole ([TEP][MIm]), were prepared and used to investigate CO_2_ capture [101]. The results indicated that the original [PMIm]Cl had low CO_2_ absorption capacity at ambient temperature and pressure, whereas the functionalized ionic liquids had a maximum CO_2_ capture capacity of approximately 0.31 mol/mol. The formulation containing 20 wt% tetraethylene pentamine-2-methylimidazole ([TEP][MIm]) exhibited the highest CO_2_ capture capacity of around 1.93 mol/mol. The synthesized ZIF-8-ionic liquids demonstrated a maximum CO_2_ capture capacity of approximately 2.22 and 2.16 mol/mol at 20 and 10 wt% ionic concentrations, respectively, with a porous ionic liquid addition of 1.0/100 g.

Two amino-functional ionic liquids were synthesized in deionized water: tetraethylenepentamine-2-methylimidazole ([TEPA][MIm]) and diethylenetriamine-2-methylimidazole ([DETA][MIm]) [102]. Two zeolite imidazolidin-8 frames (ZIF-8-W and ZIF-8-M) were prepared in methanol and deionized water. Based on the above, the porous ionic liquid ZIF-8-W/[TEPA][MIm] with the best performance and ZIF-8-W with the best environmental friendships were fabricated. The difference in adsorption capacity between ZIF-8-W and ZIF-8-M was about 36.3 mL/g; the maximum mass change rates of the two ionic liquids after immersion in ceramic and stainless-steel tubes for seven days were 0.78% and 0.29%, respectively. On the premise of ensuring fluidity, the maximum loading capacity of ZIF-8-W/[TEPA][MIm] for pure CO_2_ reached 2.22 mol/mol, and the maximum capture capacity for CO_2_ in artificial flue gas reached 2.03 mol/mol, which was 21.9% and 31.6% higher than the maximum capture capacity of pure ionic liquids, respectively. When [TEPA][MIm] in the porous ionic liquid reacts with CO_2_, it conforms to the characteristics of “zwitterions”. Note the difference in the formulation of tetraethylenepentamine-2-methylimidazole between this reference and the previous one [101].

Including the different pathways to fabricate composites, the impregnation method was used to form a hybrid composite of 1-butyl-3-methylimidazole acetate ([BMIM][Ac]) and the molecular sieve SAPO-34 [103]. This composite was used to investigate its performance on CO_2_ capture. The results showed that SAPO-34 retained its original structure after [BMIM][Ac] impregnation. The CO_2_ uptake of 1.879 mmol/g at 30 °C and 1 bar resulted in a 20.6% enhancement when compared to that of the pristine SAPO-34 (1.558 mmol/g). With an acceleration of the CO_2_ loading kinetics, the apparent mass transfer resistance for CO_2_ capture was reduced by 11.2% compared with that of the pure [BMIM][Ac]. The differential scanning calorimetry method revealed that the loaded sample had a lower CO_2_ desorption heat than that of the pure [BMIM][Ac], and the CO_2_ desorption heat of the loaded samples was between 30.6 and 40.8 kJ/mol.

As is well known, graphene has multiple uses, which include the removal of gases. Density functional theory calculations were employed to evaluate the possibility of utilizing graphene as a carrier material to increase the CO_2_ adsorption uptake of tetramethylammonium 2- hydroxypyridine salt ([TMA][HPy]) [104]. The results indicated that BN co-doped fluorinated graphene represented a carrier material, which weakens the interaction between cations and anions while enhancing the charges of oxygen and nitrogen in the anionic species of the ionic liquid. The above resulted in an increase in the CO_2_ adsorption capacity. The adsorption sites of the ionic liquids and their properties were influenced by doping atoms in the graphene, because these atoms produced alterations in the distribution of surface electrostatic potential. Compared to hydrogen-terminated graphene, fluorinated graphene was a more stable carrier material due to its higher binding energy with this ionic liquid. The higher binding energy was not derived from the direct interaction between doped atoms and the ionic liquid but, rather, from the impact of doped atoms on the electronic structure of graphene. With methanol acting as the solvent, the adsorption of the ionic liquid on various graphene surfaces was a spontaneous exothermic process.

Following the concept of a one-step synthesis procedure, an amino-functionalized imidazolium ionic liquid as an organic monodentate ligand was used in the fabrication of microporous Cu-based I-MOFs (ionic metal–organic frameworks) [105]. Precise tuning of the adsorption properties was obtained by incorporating aromatic anions, such as phenoxy, benzene carboxyl, and benzene sulfonic acid group, into the I-MOFs via an ion exchange process. The I-MOFs showed high thermal stability and uptake of 5.4 mmol/g under atmospheric conditions for the selective adsorption of CO_2_, again improving the results of using the pristine framework. The active sites of microporous Cu-MOF are the ion basic center and unsaturated metal, and electrostatic attraction and hydroxyl bonding between CO_2_ and modified functional sulfonic groups are responsible for the adsorption.

### Remarks About Adsorption and Ionic Liquids

Similar to our comment in Section 2.3, in the present case, it is also difficult, if not unrealistic, to conduct a comparison between the different systems used to remove CO_2_ from gaseous streams. In any case, Table 12 presents a series of CO_2_ uptakes onto various ionic liquid adsorption systems in order to see the variety of figures than one can obtain with the different systems.

## 4. Membranes and Ionic Liquids

**Figure 1:**
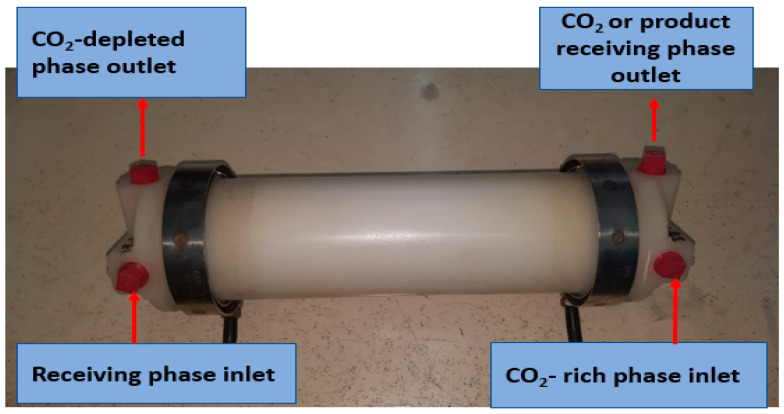
Commercial hollow fiber membrane module to investigate CO_2_ permeation phenomena. Phases entering the module in counter-current operational form (co-current form is also possible, and the way (tube or shell sides) in which the phases feed the module). Module length: 28 cm. Fiber length: 15 cm. Effective membrane area: 1.4 m^2^.

Ionic liquid-based membranes are being considered as promising materials for CO_2_ capture and/or separation due to their permeation and selectivity properties. Though the investigations in this field use a number of operational devices, one useful item is in the form of hollow fiber membrane modules (Figure 1), which allow for experiments in a continuous basis and also using different operational options.

In these membrane systems, also in reference to mixed-matrix membranes, the ionic liquid is supported into the micropores of the membrane or formed part of the membrane as a consequence of the membrane casting process.

A composite membrane composed of ethanolamine-based ionic liquids and poly(vinyl alcohol) (PVA) was developed to investigate its usefulness for CO_2_ capture and CO_2_/CH_4_ separation [106]. From all the formulations investigated, the results showed that the higher CO_2_ permeabilities were obtained with [m-2HEA][Pr] and [2HEA][Pr] (165 and 185 barrer, respectively). The investigation also concluded that the [BHEA][Bu] formulation produced membranes with the highest CO_2_/CH_4_ selectivity (241) and stability (−4.2 barrer/h). The above formulations corresponded to the respective compositions of N-methyl-2-hydroxyethylammonium propionate, 2-hydroxyethylammonium propionate and bis-2-hydroxyethylammonium butanoate.

Supported ionic liquid membranes containing aprotic N-heterocyclic anion ionic liquids in an inorganic inert support exhibit CO_2_/N_2_ permselectivity values of 640 at 35 °C and 0.03 bar CO_2_ [107], which represents conditions similar to post-combustion carbon capture (PCCC) from a natural gas power plant. A Fickian model fitted to the experimental data estimated CO_2_ permeability, in direct air capture conditions, of 10,400 barrer and a CO_2_/N_2_ permselectivity of 4000 for the best-performing ionic liquid (triethyl(octyl)phosphonium 4-bromopyrazolide ([P_2228_][4-BrPyra])). The most important criterion for high selectivity is a large equilibrium constant for binding between the ionic liquid and CO_2_, which resulted in high carbon dioxide solubility. Ionic liquids with smaller molar volumes and with no fluoroalkyl chains enhanced N_2_ rejection. Low viscosity and high ionic liquid molar density also enhanced CO_2_/N_2_ permselectivity and CO_2_ capture.

The triphenylbenzene-dimethoxyterephthaldehyde-covalent organic framework (TPB-DMTP-COF) was modified with an imidazolium-based ionic liquid ([EMIM][NTf_2_]) to overcome disadvantages presented by the use of COFs, and then the mixed-matrix membranes were prepared by introducing IL@COF into PIM-1 (PIM: polymers of intrinsic microporous) [108]. The ionic liquid modification resulted in a material TPB-DMTP-COF with better CO_2_ affinity and narrowed pore size but also further improved the interfacial compatibility among PIM-1 and TPB-DMTP-COF. Therefore, the introduction of IL@COF into the PIM-1 membrane can lead to an increase in the CO_2_/N_2_ separation properties of the resulting membranes. Among the various formulations, 3.0 wt% IL@COF/PIM-1 mixed-matrix membranes show CO_2_ permeability and CO_2_/N_2_ selectivity of near 9138 barrer and 20.2, respectively. Such separation performance exceeds the 2008 Robeson upper bound.

Mixed-matrix membranes (MMMs) based on Pebax^®^1657 were obtained utilizing synthetic silico-metallic mineral particles (SSMMPs) functionalized with [BMIM][NTf_2_] ionic liquid [109]. For the membrane fabrication, an ethanol/water mixture was used as a solvent, with the SSMMP/ionic liquid content varying from 0.5 to 20% by the weight of the polymer. Gas permeability and ideal selectivity were measured at 25 °C and different pressures, ranging from 1 to 7 bar. The presence of SSMP/ionic liquid reduced the flexibility of the PEO chains, resulting in better CO_2_/N_2_ and CO_2_/CH_4_ selectivities. In the CO_2_/N_2_ system, the highest selectivity was reached at lower filler concentrations, gradually decreasing as the filler load increased. The MMM-0.5 wt% formulation resulted in the highest selectivity of near 92. The membrane CO_2_ permeability increases with an elevated filler content, from 84.21 for pure Pebax^®^1657 to 1927 barrer for MMM-20 wt% at 4 bar. The majority of the obtained MMMs have separation performances above the 2008 Robeson upper bound. In the CO_2_/CH_4_ system, the membranes performed below Robeson’s curve. However, the addition of 5%, 10%, and 20 wt% of SSMMP/ionic liquid resulted in performances very close to the 1991 Robeson upper bound.

The use of double-network gels can also be used in the removal of toxic components from gas streams. Pebax 1657, composed of a semicrystalline polyamide block, was the first network in the ion gel membrane [110]. The usefulness of this block is that it served to form a network to dissipate the loaded energy. In the present investigation, the polyamide block was interpenetrated with a tetra-PEG hidden-length network, which is highly stretchable and compatible with the ionic liquid 1-ethyl-3-methylimidazolium tricyanomethanide. This high mechanical strength allowed researchers to develop an ion gel membrane, retaining 92.5 wt% of the ionic liquid with the convenient performance towards CO_2_ capture. The as-formed ion gel membrane had a CO_2_ permeability and CO_2_/N_2_ permselectivity of 3100 barrer and 43, respectively. The permeability of the toxic gas across the ion gel membrane increased with the increase in the relative humidity without compromising the CO_2_/N_2_ permselectivity (Table 13). However, the increase in the temperature from 30 to 80 °C increases CO_2_ permeability but decreases CO_2_/N_2_ permselectivity. The stability in the CO_2_ permeation was maintained for more than 10 days under a relative humidity of 80% at 80 °C. The above data fulfil the requirements for the utilization of this membrane in the capture of CO_2_ from flue gases released by coal-fired power plants.

A thin-film composite mixed-matrix membrane was fabricated using an ionic liquid and zeolitic imidazolate framework-8 (ZIF-8) dispersed in a polymerizable ionic liquid (PIL) matrix for CO_2_ separation [111]. A PIL comb copolymer, i.e., poly(1-allyl-3-methylimidazolium bis(trifluoromethanesulfonyl)imide-co-poly(ethylene glycol) methyl ether methacrylate) (poly(AMIM-TFSI-co-POEM), PAP), was synthesized via free radical polymerization. The ionic liquid 1-ethyl-3-methylimidazolium bis(trifluoromethylsulfonyl)imide ([EMIM][TFSI]) served to promote CO_2_ solubility, while 60 nm sized ZIF-8 nanoparticles acted as a size-sieving agent. The crystalline and pore structures of ZIF-8 were maintained in the composite membrane, with no significant PAP/ionic liquid infiltration into the pores of ZIF-8. The 600 nm thick TFC-MMM with the PAP/IL/ZIF-8 ternary system exhibited a CO_2_ permeability of 1017 GPU, CO_2_/N_2_ selectivity of 33, and CO_2_/CH_4_ selectivity of 13, values which fulfilled the commercial criteria required for post-combustion CO_2_ capture.

Two ionic liquids were used for the preparation of cellulose acetate (CA)–ionic liquid blend membranes. The ionic liquids were 1-butyl-3-methylimidazolium hydrogen sulfate ([BMIM][HSO_4_]) and choline glycine ([Cho][Gly]), as they present adequate CO_2_ dissolution properties [112]. Several composite membranes were prepared through the solvent casting technique, showing that the ionic liquids strongly interacted with the C=O groups of cellulose acetate, which exhibited high affinity with CO_2_. In the case of the first ionic liquid, a reduction in the available sites that allow for strong intermolecular interactions with CO_2_ resulted in a decrease in the capture of this gas compared to that of pure cellulose acetate. In the case of choline glycine, the reduction was compensated by the presence of specific groups in the ionic liquid, which presented high affinity towards CO_2_. It is concluded that the CA-[Cho][Gly] mixed membranes had better CO_2_ removal capacity, in addition to other advantages, such as non-toxicity and low cost.

In this investigation [113], 3D-printable materials based on ad hoc synthesized photocurable imidazolium ionic liquids, 1-(4-vinylbenzyl)-2-methyl-3-butylimidazolium bis(trifluoromethanesulfonyl)imide ([C_4_vBMIM][NTf_2_]) and 1-(4-vinylbenzyl)-2-methyl-3-3,3-dimethylbutylimidazolium bis(trifluoromethanesulfonyl)imide ([(CH_3_)_2_C_4_ vBMIM][NTf_2_]), were examined. Polymerization was carried out on formulations containing a crosslinking monomer (PEGDA). The investigation confirmed the reactivity of both formulations, making them suitable for the digital light processing 3D-printing technique. Membranes were then tested through high-pressure CO_2_ loading analysis to estimate their capture efficiency, comparing the results with the standard room-temperature ionic liquid counterpart. The results showed an increase in the gas removal when the pressure increased from 0 to 40 bar at 25 °C. Complex gyroid-like structures incorporating the synthesized ionic liquids were 3D printed, showing the ability of these materials to be processed with 3D-printing technology while maintaining the CO_2_ capture performance of ionic liquids.

In order to avoid some drawbacks (poor mechanical properties, limited stability or low gas permeability), an investigation to prepare double-network ion gel membranes with both high strength and remarkable gas separation performance was performed [114]. Whereas the membrane with the poly[VEIM][TFSI]:[EMIM][DCA] (70 wt%) formulation presented a CO_2_ permeability of 464 barrer and CO_2_/N_2_ separation of 63, surpassing the 2008 upper bound, the membrane formed of poly[VEIM][TFSI]:[EMIM][TFSI] (80 wt%) did not reach this upper bound (PCO_2_ = 866 barrer and CO_2_/N_2_ separation of 31). In the above formulations, [VEIM][TFSI] and [EMIM][DCA] stand for 1-vinyl-3-ethylimidazole bis (trifluoromethanesulfonyl) imide and 1-ethyl-3-methylimidazolium dicyanamide, respectively.

Imidazolium-based ionic liquid monomers (IL1: 1-cyanomethyl-3-vinylimidazolium bromide and IL2: 1-carboxymethyl-3-vinylimidazolium bromide) were co-polymerized with two functionalized monomers of acrylamide (AM) and butyl acrylate (BA) [115], and the influence of the chemical structures for PIL-based copolymers on their performances of derived membranes for CO_2_/N_2_ separation was investigated. These PIL-based composite membranes were formed coating the copolymer solutions on the surface of a commercial polysulfone (PSF) membrane. It was experimentally observed that the best results were yielded from the use of the membrane containing IL-1 and acrylamide, with a CO_2_ permeability of 76 GPU and CO_2_/N_2_ selectivity (53), resulting in 262% and 61% increases when compared with the use of single PSF membranes.

Two amino-functionalized PILs (1-cyanomethyl-3-vinylimidazolium bromide and (4-aminoethyl-1-vinylimidazolium bromide) hydrobromide) were used in the fabrication of composite membranes. In this fabrication, the formed PIL-based solutions were cast on the superior part of polysulfone ultrafiltration membranes, aiming to investigate the influence of the chemical structures on the separation performance of the NH_2_-carrier-based membranes [116]. The membranes presented experimental CO_2_/N_2_ selectivity of near 174 when the mobile carrier of 2-(1-piperazinyl) ethylamine sarcosine (PZEA-Sar) was introduced into the amino-functionalized PIL-based membranes. The above mixture had a synergistic influence from both fixed-site and mobile carriers and, thus, promoted CO_2_-facilitated transport.

The introduction of graphite-like phase carbon nitride (g-C_3_N_4_) nanosheets and interaction networks (PGCNs) built up, taking advantage of the dipole-level quadrupole moment interactions between the ether-oxygen groups contained in polyethylene oxide (PEO) and CO_2_ [117]. Membrane surface defects were compensated by coating the ionic liquid on the surface of the membrane, creating a PEO@g-C_3_N_4-_supported liquid membrane (PGCN/ILX SILM). The results (Table 14) showed that the membrane containing [EMIM][ACO] 30 wt% presented the best overall performance.

### Remarks About Membranes and Ionic Liquids

Obviously, comparisons of the various ionic liquid–membrane system are also subjective; thus, as in the above technologies, these authors opted to show (Table 15) results derived from the investigations for the readers’ benefit. The results on CO_2_/N_2_ selectivity are included because this pair is the most extensively studied using this technology.

## 5. Conclusions

**Figure 2:**
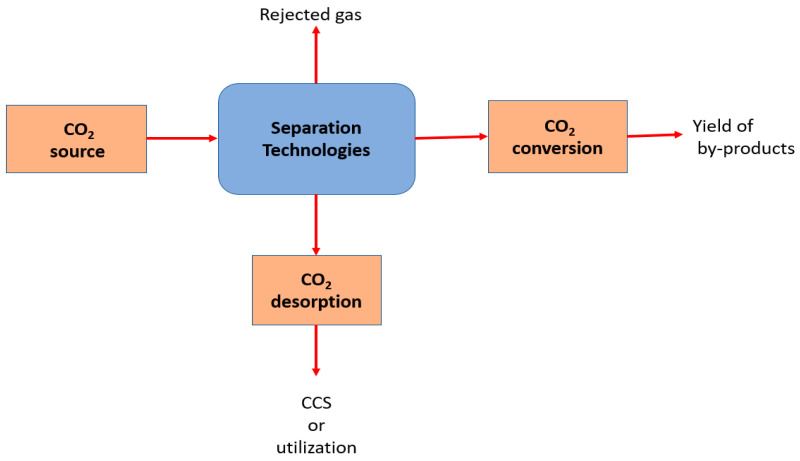
Scheme of different types of carbon dioxide capture/separation processes.

This work reviewed the utilization of different separation technologies (absorption, adsorption and membranes) together with the use of ionic liquids to improve CO_2_ capture. With the logical operational variables among these technologies, a general overview (Figure 2) can be established. The CO_2_ stream feeding any of the technologies allows for the capture of this gas and the rejection of the CO_2_-depleted gas stream (containing other non-captured toxic gases). Two options are accomplished: In one of them, captured CO_2_ is desorbed from the corresponding material or solution for either capture and storage (CCS) or further utilization of the gas. In the second (or first option depending of the point of view), CO_2_ is converted into a suitable by-product at the time of capture.

As alternatives to CCS (CO_2_ capture and storage), CCU (CO_2_ capture and utilization) and ICCU (integrated CO_2_ capture and utilization) are key strategies to develop zero or even negative carbon processes. Current investigations on CO_2_ capture are the first step for its industrial applications, but to afford the scaling-up of these investigations, there are some points that need to be resolved, i.e., the viscosity of ionic liquids and, sometimes, relatively low mass transfer. The industrialization of these various strategies depends on various issues: cost-effective CO_2_ ionic liquid adsorbents, efficient conversion protocols, stable materials (ionic liquids, metal–organic frameworks, membranes, etc.) as well as suitable equipment to achieve this capture in an industrialized form. Also, and no less important, we need to investigate these systems on real gas streams to evaluate the real performance of these proposals in real situations.

Challenges to consider (without any particular order) include the following:

- Development of polymeric membranes for CO_2_ capture and separation, since these membranes present certain advantages, i.e., low energy demand and small equipment volume.
- Enhancement of CO_2_ separation through the development of poly(ionic liquids) containing amino groups due to the tunability of the cation and anion, as well as the high CO_2_ affinity towards the amino groups.
- Development of ionic metal–organic frameworks (I-MOFs) and other materials of interest, like graphene and ionic liquids.
- Development of porous ionic liquids, formed of an ionic liquid and a suitable solid porous material, which can improve CO_2_ capture and storage.
- The use of 3D-printing technology in the development of complex gyroid-like structures, incorporating ionic liquids.
- Usage of highly efficient catalysts for the conversion of low-partial-pressure CO_2_ under mild conditions.
- Development of materials to improve low-concentration CO_2_ capture.
- Replacement of aqueous amine solutions for the capture of CO_2_ in post-combustion technologies. The application of functionalized ionic liquids, i.e., amino acid ionic liquids, in carbon dioxide (CO_2_) capture seemed to be promising; however, some questions needed further investigations, like analysis of the interaction of amino groups in these amino acid ionic liquids with CO_2_, evaluation of the energy consumption of CO_2_ capture, improvement in their (solvents) high viscosity and low mass transfer.
- Improvements in amine solvent regeneration and decrease in degradation of the amines with absorption/regeneration cycles.
- Development of functionalized solvents with improved characteristics: low volatility, high mass capacity, and low energy consumption for CO_2_ capture.
- Utilization of chemisorption with biphasic solvents, being this a low-energy strategy for separating CO_2_ from industrial flue gas. Investigation of the solvent degradation and changes in water content can be difficult in terms of the phase-splitting performance.

## Figures and Tables

**Table 1:** Cationic and anionic moieties used in the calculations.

Cation	Anion
1-Allyl-3-methylimidazolium [AllylMIM] 1-Butyl-3-methylimidazolium [BMIM] 1-Butyl-3-methylpyridinium [BMP] 1-Butyl-1-methylpyrrolidinium [BMPYR] 1,2-Dimethyl-3-propylimidazolium [DMPIIM] 1-Ethyl-3-methylimidazolium [EMIM] 1-Hexyl-3-methylimidazolium [HMIM] 1-Methyl-3-octylimidazolium [OMIM] 1-Methyl-3-propylimidazolium [PMIM]	Bis(trifluoromethylsulfonyl)imide [NTf2] Dicyanimide [DCA] Hexafluorophosphate [PF6] Triflate [Triflate] Tetrafluoroborate [BF4] Ethyl sulphate [ESO_4_]

Adapted from [22].

**Table 2:** CO_2_ absorption (x_2_) using different ionic liquids.

Pressure, MPa	[1MeimPS][TRp]	[1MeimPS][Tyr]	[1MeimPS][His]
1.0 2.0 3.0 4.0	0.34 0.43 0.51 0.59	0.38 0.48 0.53 0.55	0.51 0.63 0.71 0.73

**Table 3:** CO_2_ uptakes using different acetate-based ionic liquids.

Ionic Liquid	CO_2_ Uptake, mol/kg
HIIL CPIIL DImIIL	6.2 4.5 16.8

Adapted from [42]. Time: 45 min. Pressure: atmospheric. HIIL: 1,3-bus(2-hydroxyethyl)-1H-imidazol-3-ium acetate. CPIIL: 1,3-bis(2-(3-chloro-2-hydroxypropoxy)ethyl)-1H-imidazol-3-ium acetate. DImIIL: 3,3-((1H-2-(3-1-H-imidazole-3-ium-1,3-diyl)bis(ethane-1,2-diyl))bis(2-hydroxypropane-3,1-diyl)) bis(1-methyl-1H-imidazol-3-ium) acetate dichloride.

**Table 4:** Approximate CO_2_ uptakes at various temperatures and CO_2_ partial pressures.

Temperature, °C	^a^ mol CO_2_/kg PEI + IL	^b^ mol CO_2_/PEI + IL
25 40 60	4.7 4.4 4.1	5.0 4.6 4.5

Adapted from [57]. X_W(PEI)_:0.3. **^a^** At 0.3 bar. **^b^** At 10 bar.

**Table 5:** CO_2_ uploading at various MMA/MDA ratios.

MMA/MDA Ratio	CO_2_ Uploading, mol/mol
1/1 2/1 3/1 4/1	0.42 0.47 0.62 0.62

Adapted from [67]. Stirring rate: 800 min^−1^. Flow: 98 L/h·kg. Temperature: 25 °C.

**Table 6:** CO_2_ uptake at various v% DMSO/v% water ratios.

v% DMSO/v% Water Ratio	CO_2_ Uptake, mol/mol
Pure DMSO 70/30 50/50 30/70 Pure water	0.45 1.03 1.22 1.45 1.86

Adapted from [75]. Experiments on pure CO_2_.

**Table 7:** CO_2_ uptake values using ionic liquid absorption from monophasic systems.

Absorbent	CO_2_ Uptake, mol/mol	Reference
[P_4442_][Hy] MDEA+ [BMIM][TFO] [P_66614_][3-HMPz] [N_1111_][Maba] + PMDETA [N_1111_][Eaca] + PMDETA [DBUH][Maba] [DBNH][Maba] [N_1111_][β-iPrNH-Ala] [BMIM][Tz] [DBNH][2-OP]	1.90 2 < x < 2.5 1.29 1.4 1.0 0.58 0.78 0.78 1.02 1.4	[23] [35] [39] [47] [47] [48] [48] [49] [53] [58]

**Table 8:** CO_2_ uptake values using ionic liquid absorption from biphasic systems.

System	CO_2_ Uptake, mol/mol	Reference
[TEPAH][CPh]/EG/DGDE [TEPAH][CPh]/EG/DGDE [BMIM][NTf_2_]/MMEA/MDEA [DMAPA][TZ]/water/NHD [DMAPA][TZ]/water/PC [DETA][SER]/water/NHD [N_2221_][Arg]/water/DMSO	1.88 (40 °C) 1.83 (60 °C) 0.62 1.14 0.95 1.26 1.45	[62] [62] [67] [69] [69] [70] [75]

**Table 9:** CO_2_ capture by PIL-substituted imidazolium cations at various pressures.

PIL-alkyl	Pressure, bar (CO_2_ Uptake, mg/g)
PIL-me PIL-n-butyl PIL-n-hexyl	1.1(9.6), 10.0(159.28) 0.9 (4.30), 10.1 (111.42) 1.0 (4.34), 9.9 (81.81)

Adapted from [79]. Temperature: 35 °C.

**Table 10:** The various gas mixtures used in the removal of CO_2_ by [TETA][Lys]@ZIF-8.

Mixture	Gas and Elution Time
CO_2_/C_2_H_2_ (50/50) CO_2_/C_2_H_4_ (1/99) CO_2_/CH_4_ (15/85) CO_2_/N_2_ (10/90) CO_2_/CH_4_/C_2_H_6_/C_3_H_8_ (15/85/4/1) CO_2_/N_2_/O_2_ (1/79/20)	CO_2_ 6 min, C_2_H_2_ 2.5 min CO_2_ 230 min, C_2_H_4_ < 1 min CO_2_ 30 min, CH_4_ < 1 min CO_2_ 60 min, N_2_ < 1 min CO_2_ 20 min, CH_4_ < 1 min, C_2_H_6_ < 1 min, C_3_H_8_ 5 min CO_2_ 140 min, N_2_ < 1 min, O_2_ < 1 min

Adapted from [84]. Temperature: 25 °C. Pressure: 100 kPa.

**Table 11:** CO_2_ uptake at various relative humidities.

Relative Humidity, %	CO_2_ Uptake, mmol/g
0 15 25 35 55	1.8 2.4 2.1 1.9 1.6

Adapted from [90]. Temperature: 25 °C. 1 v% CO_2_ containing gas mixture balanced with N_2_.

**Table 12:** CO_2_ uptakes onto ionic liquid adsorbent systems.

System	CO_2_ Uptake, mmol/g	Reference
[BMIM][AC]-biochar C:E:M1:P2_80%_ SBA-15@DIL_2FeCl_4_ PDVN-5 [TMGH][IM]@ZIF-8 [H_2_Pi][HSO_4_]_2_-Mg(15)-HKUST-1 [EMIM][2-CNpyr]/diethylene glycol Cu-AFIL-M-SB	1.47 0.53 1.30 0.22 0.40 6.05 0.66 5.40	[78] [80] [82] [83] [86] [89] [98] [105]

**Table 13:** Influence of the relative humidity on CO_2_ permeation and CO_2_/N_2_ permselectivity.

Relative Humidity, %	CO_2_, Barrer	CO_2_/N_2_
0 20 40 60 80	1450 1600 1800 1900 2000	26 26 26 26 26

Adapted from [110]. Feed gas: 50/50 mol/mol CO_2_/N_2_. Temperature: 80 °C. Atmospheric pressure.

**Table 14:** Performance of supported liquid membranes (SLMs) derived from g-C_3_N_4_ and PGCN.

SLM	Ionic Liquid, wt%	GPU	CO_2_/N_2_ Separation
PGCN/[EMIM][AcO]	20 30 40	1577 1396 976	32 58 50
PGCN/[BMIM][BF_4_]	20 30 40	990 937 839	38 39 34

Adapted from [117]. Temperature: 25 °C. Pressure: 25 bar. [EMIM][AcO]: 1ethyl-3methylimidazole acetate. [BMIM][BF_4_]: 1-ethyl-3-ethylimidazole tetrafluoroborate.

**Table 15:** CO_2_ permeabilities and CO_2_/N_2_ selectivity using ionic liquid membranes.

Ionic Liquid	CO_2_ Permeation, Barrer	CO_2_/N_2_	Reference
[2HEA][Pr] [P_2228_][4-BrPyral] [EMIM][NTF_2_] [BMIM][NTf_2_] [EMIM][NTf_2_] [VEMIM][TFSI]:[EMIM][DCA]	185 10,400 ^a^ 9138 192 610 464	640 20 92 33 63	[106] [107] [108] [109] [111] [114]

^a^ Modeled.

## References

[1] Chen Z., Yuan B., Zhan G., Li Y., Li J., Chen J., Peng Y., Wang L., You C., Li J. (2022). Energy-efficient biphasic solvents for industrial carbon capture: Role of physical solvents on CO_2_ absorption and phase splitting. Environ. Sci. Technol..

[2] Xing L., Wei K.X., Li Q.W., Wang R.J., Zhang S.H., Wang L.D. (2020). One-step yynthesized SO_4_^2−^/ZrO_2_-HZSM-5 solid acid catalyst for carbamate decomposition in CO_2_ capture. Environ. Sci. Technol..

[3] Yuan B., Zhan G., Chen Z., Li Y., Wang L., You C., Li J. (2022). Intrinsic insight of energy-efficiency optimization for CO_2_ capture by amine-based solvent: Effect of mass transfer and solvent regeneration. Int. J. Greenhouse Gas Control.

[4] Alvarez-Guerra M., Irabien A. (2011). Design of ionic liquids: An ecotoxicity (*Vibrio fischeri*) discrimination approach. Green Chem..

[5] Jordan A., Gathergood N. (2015). Biodegradation of ionic liquids-a critical review. Chem. Soc. Rev..

[6] Mena I.F., Diaz E., Rodriguez J.J., Mohedano A.F. (2022). An overview of ionic liquid degradation by advanced oxidation processes. Crit. Rev. Environ. Sci. Technol..

[7] Cho C.W., Pham T.P.T., Zhao Y., Stolte S., Yun Y.S. (2021). Review of the toxic effects of ionic liquids. Sci. Total Environ..

[8] Shah F., An R., Muhammad N. (2020). Editorial: Properties and applications of ionic liquids in energy and environmental science. Front. Chem..

[9] Kaur G., Kumar H., Singla M. (2022). Diverse applications of ionic liquids: A comprehensive review. J. Mol. Liq..

[10] Mušović J., Tekić D., Marić S., Jocić A., Stanković D., Dimitrijević A. (2024). Sustainable recovery of cobalt and lithium from lithium-ion battery cathode material by combining sulfate leachates and aqueous biphasic systems based on tetrabutylphosphonium-ionic liquids. Sep. Purif. Technol..

[11] Oke E.A., Ijardar S.P. (2021). Insights into the separation of metals, dyes and pesticides using ionic liquid based aqueous biphasic systems. J. Mol. Liq..

[12] Alguacil F.J., Robla J.I. (2023). Recent work on the recovery of rare earths using ionic liquids and deep eutectic solvents. Minerals.

[13] Chen S., Wang N., Zhang H., Qiu M., Shi L., Xia Y., Zhang J., Huang Y., Cheng F., Gu P. (2024). Ionic liquid-functionalized metal-organic frameworks/covalent-organic frameworks for CO_2_ capture and conversion. Ind. Eng. Chem. Res..

[14] Pandya I., El Seoud O.A., Assiri M.A., Kumar Kailasa S., Malek N.I. (2024). Ionic liquid/metal organic framework composites as a new class of materials for CO_2_ capture: Present scenario and future perspective. J. Mol. Liq..

[15] Wu W.-Y., Zhang M., Wang C., Tao L., Bu J., Zhu Q. (2024). Harnessing ash for sustainable CO_2_ absorption: Current strategies and future prospects. Chem.-Asian J..

[16] Lebedeva O., Kultin D., Kustov L. (2024). Polymeric ionic liquids: Here, there and everywhere. Eur. Polym. J..

[17] Luo W., Wang C., Jin M., Li F., Li H., Zhang Z., Zhang X., Liang Y., Huang G., Zhou T. (2024). Research progress on nanoconfined ILs in two-dimensional composite membranes for CO_2_ capture. Sep. Purif. Technol..

[18] Afkhamipour M., Seifi E., Esmaeili A., Shamsi M., Borhani T.N. (2024). Comparison of CO_2_ absorption in DETA solution and [bmim]-[PF6] using thermodynamic and process modelling. Fuel.

[19] Ali M., Sarwar T., Mubarak N.M., Karri R.R., Ghalib L., Bibi A., Mazari S.A. (2024). Prediction of CO_2_ solubility in ionic liquids for CO_2_ capture using deep learning models. Sci. Rep..

[20] Amin P., Memarian A., Repo E., Andersson M., Mansouri S.S., Zendehboudi S., Rezaei N. (2024). Mathematical modeling of dispersed CO_2_ dissolution in ionic liquids: Application to carbon capture. J. Mol. Liq..

[21] Attri P., Koga K., Razzokov J., Okumura T., Kamataki K., Nozaki T., Shiratani M. (2024). Plasma-ionic liquid-assisted CO_2_ capture and conversion: A novel technology. Appl. Phys. Express.

[22] Chauhan R., Sartape R., Mishra R., Shah J.K., Singh M.R. (2024). High-throughput measurements of CO_2_ permeance and solubility in ionic liquid reveal a synergistic role of ionic interactions and void fractions. Chem. Eng. J..

[23] Chen T., Sun Z., Guo Y., Xu Y. (2024). Does the active hydrogen atom in the hydantoin anion affect the physical properties, CO_2_ capture and conversion of ionic liquids?. Phys. Chem. Chem. Phys..

[24] Deepan Kumar M., Jaccob M. (2024). Revealing the non-covalent interactions existing between CO_2_ and succinimide-based ionic liquid: A DFT exploration. J. Mol. Liq..

[25] Dongare S., Coskun O.K., Cagli E., Stanley J.S., Mir A.Q., Brower R.S., Velázquez J.M., Yang J.Y., Sacci R.L., Gurkan B. (2024). Key experimental considerations when evaluating functional ionic liquids for combined capture and electrochemical conversion of CO_2_. Langmuir.

[26] El-Nagar R.A., Elaraby A., Nessim M.I., Ghanem A. (2024). Designed imidazolium-based ionic liquids to capture carbon dioxide from natural gas. J. Mol. Liq..

[27] Karim H., Sardar S., Bibi H., Perveen F., Arfan M., Mumtaz A. (2024). Effect of the anionic counterpart of amino acid based ionic liquids upon efficient CO_2_ capture: A correlation of experimental and DFT study. J. Mol. Liq..

[28] Keller A.N., Kelkar P., Baldea M., Stadtherr M.A., Brennecke J.F. (2024). Thermophysical property prediction of anion-functionalized ionic liquids for CO_2_ capture. J. Mol. Liq..

[29] Khosromanesh M.R., Rashidi H. (2024). An insight on reaction kinetics of amino-acid-functionalized ionic liquid monoethanolamine glycinate for CO_2_ capture. Sep. Sci. Technol..

[30] Kodirov A., Abduvokhidov D., Mamatkulov S., Shahzad A., Razzokov J. (2024). The absorption mechanisms of CO_2_, H_2_S and CH_4_ molecules in [EMIM][SCN] and [EMIM][DCA] ionic liquids: A computational insight. Fluid Phase Equilibria.

[31] Liu X., Wang Y., He M. (2024). Quantitative analysis method for absorption mechanism and energy consumption of CO_2_ capture by amino acid ionic liquids. Process Saf. Environ. Prot..

[32] Madugula A.C.S., Jeffryes C., Henry J., Gossage J., Benson T.J. (2024). A simulation-based model studying monoethanolamine and aprotic heterocyclic anion ionic liquid (AHA-IL) mixtures for carbon capture. Comput. Chem. Eng..

[33] Mao L., Chen Y., Zhang R., Yang C., Zhou Y., Zhang G. (2024). Cost-effective preparation of carbonic anhydrase with superior performance for assisting amine and amino acid ionic liquid blends in CO_2_ absorption and desorption. ACS Sustain. Chem. Eng..

[34] Mohammed S., Eljack F., Kazi M.-K., Atilhan M. (2024). Development of a deep learning-based group contribution framework for targeted design of ionic liquids. Comput. Chem. Eng..

[35] Nawaz Khan S., Abbas F., Enujekwu F.M., Ullah S., Ali Assiri M., Al-Sehemi A.G. (2024). Preparation of ionic liquids amine hybrid solvents, characterization for CO_2_ absorption, and kinetic performance. J. Mol. Liq..

[36] Nakhaei-Kohani R., Amiri-Ramsheh B., Pourmahdi M., Atashrouz S., Abedi A., Mohaddespour A., Hemmati-Sarapardeh A. (2024). Extensive data analysis and modelling of carbon dioxide solubility in ionic liquids using chemical structure-based ensemble learning approaches. Fluid Phase Equilibria.

[37] Park J., Cañada L.M., Brennecke J.F. (2024). Thermal stability and CO_2_ uptake of dicationic ionic liquids containing 2-cyanopyrrolide anions. J. Chem. Eng. Data.

[38] Qin S., Dai S., Fan W., Li M., He Z., Qi J., Yang Y., Yan H. (2024). A novel ionic liquid absorbent with polyamidoamine dendrimer as cations for efficient CO_2_ absorption. J. Mol. Liq..

[39] Qiu L., Fu Y., Yang Z., Johnson A.C., Do-Thanh C.-L., Thapaliya B.P., Mahurin S.M., He L.-N., Jiang D.-E., Dai S. (2024). Surpassing the performance of phenolate-derived ionic liquids in CO_2_ chemisorption by harnessing the robust nature of pyrazolonates. ChemSusChem.

[40] Quaye E., Henni A., Shirif E. (2024). Carbon dioxide solubility in three bis tri (fluromethylsulfonyl) imide-based ionic liquids. Molecules.

[41] Shaikh A.R., Vidal-López A., Brotons-Rufes A., Pajski J.J., Zafar S., Mahmood R.A., Khan M.U., Poater A., Chawla M., Cavallo L. (2024). Amino acid ionic liquids as efficient catalysts for CO_2_ capture: A combined static and dynamic approach. Results Surf. Interfaces.

[42] Shawish I., Al Ayoubi S., Bououdina M., El-Segaey A.A., Melegy A.A., Atta A.M. (2024). New functionalized di-substituent imidazolium ionic liquids as superior faster absorbents for carbon dioxide gas. ACS Omega.

[43] Sun Q., Xiong J., Gao H., Olson W., Liang Z. (2024). Energy-efficient regeneration of amine-based solvent with environmentally friendly ionic liquid catalysts for CO_2_ capture. Chem. Eng. Sci..

[44] Syauqi A., Ningtyas J.A., Chaniago Y.D., Lim H. (2024). Techno-economic ionic liquid-based capturing, electrochemical reduction, and hydrogenation of carbon dioxide in the simultaneous production of formic acid and biomethane. J. Clean. Prod..

[45] Torkzadeh M., Moosavi M. (2024). Multiscale modeling of CO_2_ capture in dicationic ionic liquids: Evaluating the influence of hydroxyl groups using DFT-IR, COSMO-RS, and MD simulation methods. J. Chem. Phys..

[46] Wang K., Zhang Z., Wang S., Jiang L., Li H., Wang C. (2024). Dual-tuning azole-based ionic liquids for reversible CO_2_ capture from ambient air. ChemSusChem.

[47] Wen S., Wu H., Zhang X., Wang T., Xu W., Wu Y. (2024). Novel amino acid ionic liquids as messenger of multi-tertiary-amines solutions for highly efficient capture of CO_2_. Chem. Eng. Sci..

[48] Wen S., Zheng L., Zhang X., Wu Y. (2024). Unveiling protic amino acid ionic liquids for the efficient capture of carbon dioxide. Chem. Commun..

[49] Wu H., Wen S., Zhang X., Zhang S., Guo X., Wu Y. (2024). Aqueous solutions of sterically hindered amino acid ionic liquids for rapid and efficient capture of CO_2_. Chem. Eng. J..

[50] Yang A., Sun S., Mi H., Wang W., Liu J., Kong Z.Y. (2024). Interpretable feedforward neural network and XGBoost-based algorithms to predict CO_2_ solubility in ionic liquids. Ind. Eng. Chem. Res..

[51] Yang A., Sun S., Su Y., Kong Z.Y., Ren J., Shen W. (2024). Insight to the prediction of CO_2_ solubility in ionic liquids based on the interpretable machine learning model. Chem. Eng. Sci..

[52] Yang X., Zhu C., Fu T., Ma Y. (2024). A novel synergistic intensification method for CO_2_ absorption by metal salt and task-specific ionic liquids hybrid solvent in microchannel reactor. Int. J. Heat Mass Transf..

[53] Ye X., Ruan J., Chen L., Qi Z. (2024). Structure effects of imidazolium ionic liquids on integrated CO_2_ absorption and transformation to dimethyl carbonate. J. Environ. Chem. Eng..

[54] Yin H., Ma C., Duan Y., Shi S., Zhang Z., Zeng S., Han W., Zhang X. (2024). Thermodynamic modeling and process evaluation of advanced ionic liquid-based solvents for CO_2_/CH_4_ separation. Chem. Eng. J..

[55] Yoon B., Chen S., Voth G.A. (2024). On the key influence of amino acid ionic liquid anions on CO_2_ capture. J. Am. Chem. Soc..

[56] Zhang Q., Bahamon D., Alkhatib I.I.I., Zhang R., Liu Z., Liu H., Xu C., Vega L.F., Meng X. (2024). Molecular insights into the CO_2_ absorption mechanism by superbase protic ionic liquids by a combined density functional theory and molecular dynamics approach. J. Mol. Liq..

[57] Zhao Y., Wang X., Li Z., Wang H., Zhao Y., Qiu J. (2024). Understanding the positive role of ionic liquids in CO_2_ capture by poly(ethylenimine). J. Phys. Chem. B.

[58] Zhu X., Liu S., Shang R., Chen X., Xu Y., Guo Y., Ling B. (2024). Understanding on the structure of novel hydroxypyridine anion-based ionic liquids and their effect on CO_2_ absorption behavior. J. Environ. Chem. Eng..

[59] Sun X., Zeng S., Li G., Bai Y., Shang M., Zhang J., Zhang X. (2024). Selective CO_2_ separation through physicochemical absorption by triazole-functionalized ionic liquid binary absorbents. AIChE J..

[60] Albertsson P.A. (1958). Partition of proteins in liquid polymer–polymer two-phase systems. Nature.

[61] Gutowski K.E., Broker G.A., Willauer H.D., Huddleston J.G., Swatloski R.P., Holbrey J.D., Rogers R.D. (2003). Controlling the aqueous miscibility of ionic liquids: Aqueous biphasic systems of water-miscible ionic liquids and water-structuring salts for recycle, metathesis, and separations. J. Am. Chem. Soc..

[62] Zhong X., Kong W., Dong Z., Yang K., Song T., Wang T., Fang M., Li W., Li S. (2024). A novel ILs biphasic absorbent with low regeneration energy consumption for CO_2_ capture: Screening of phase separation regulators and mechanism study. Chem. Eng. J..

[63] Wang R., Qi C., Jian Z., Zhao H., Zhang P., An S., Li Q., Wang L. (2024). Development of dual-functionalized ionic liquids for biphasic solvents: Enhancing the CO_2_ absorption through two-stage reaction and promoting the energy-saving regeneration. Fuel.

[64] Tiwari S.C., Pant K.K., Upadhyayula S. (2024). Kinetics study and modeling of CO_2_ capture in new class dual-functionalized ionic liquid blend methyl diethanolamine absorbents. Ind. Eng. Chem. Res..

[65] Tiwari S.C., Pant K.K., Upadhyayula S. (2024). CO_2_ Absorption in a dual-functionalized ionic liquid-blended N-methyldiethanolamine aqueous system: A thermodynamics study. Energy Fuels.

[66] Nguyen W., Balchandani S., Mandal B., Henni A. (2024). Modeling gas–liquid equilibrium of carbon dioxide in mixtures of (1-butyl-3-methyl-imidazolium acetate + 1-(2-aminoethyl) piperazine + water) and (1-butyl-3-methyl-imidazolium acetate + bis (3-aminopropyl) amine + water) by electrolyte non-random two liquid (e-NRTL) model. Int. Commun. Heat Mass Transf..

[67] Meng F., Han S., Lin L., Li J., Chen K., Jiang J. (2024). Process optimization and mechanism study of ionic liquid-based mixed amine biphasic solvents for CO_2_ capture in biogas upgrading procedure. Front. Environ. Sci. Eng..

[68] Mao J., Ci Y., Liu J., Li C., Yang W., Yun Y., Liu G., Li M., Wang M. (2024). Experimental and theoretical investigation of an ionic liquid-based biphasic solvent for post-combustion CO_2_ capture: Breaking through the “trade-off” effect of viscosity and loading. Chem. Eng. J..

[69] Mao J., Li C., Yun Y., Liu J., Yang W., Li M., Wang L., Li C., Liu W. (2024). Biphasic solvents based on dual-functionalized ionic liquid for enhanced post-combustion CO_2_ capture and corrosion inhibition during the absorption process. Chem. Eng. J..

[70] Liu J., Mao J., Yun Y., Wang M., Liu G., Li C., Yang W., Li M., Liu Z. (2024). An amino acid ionic liquid-based biphasic solvent with low viscosity, small rich-phase volume, and high CO_2_ loading rate for efficient CO_2_ capture. Sep. Purif. Technol..

[71] Li J., Zhao Y., Zhan G., Xing L., Huang Z., Chen Z., Deng Y., Li J. (2024). Integration of physical solution and ionic liquid toward efficient phase splitting for energy-saving CO_2_ capture. Sep. Purif. Technol..

[72] Jiang W., Gao G., Gao X., Xu B., Wu F., Li X., Zhang L., Luo C. (2024). Water effect on CO_2_ absorption mechanism and phase change behavior in [N1111][Gly]/EtOH anhydrous biphasic absorbent: In density functional theory and molecular dynamics view. Chem. Eng. J..

[73] Chen W., Chen M., Jiang B., Lei T., Zhang F., Zhang Z. (2024). The improvement of ionic liquids on CO_2_ capture with biphasic absorbents. Chem. Eng. J..

[74] Chen M., Chen W., Jiang B., Huang Y., Lei T., Zhang F., Wu Y. (2024). Functional ionic liquid as phase separation trigger in biphasic absorption of CO_2_. Chem. Eng. J..

[75] Bera N., Sardar P., Samanta A.N., Sarkar N. (2024). Arginine-based ionic liquid in a water-DMSO binary mixture for highly efficient CO_2_ capture from open air. Energy Fuels.

[76] Atlaskina M.E., Kazarina O.V., Petukhov A.N., Atlaskin A.A., Tsivkovsky N.S., Tiuleanu P., Malysheva Y.B., Lin H., Zhong G.-J., Lukoyanov A.N. (2024). Amino acid-based ionic liquid as a promising CO_2_ sorption increasing agent by aqueous MDEA solution. J. Mol. Liq..

[77] Sun X., Zeng S., Peng K., Liu W., Bai L., Jiang Y., Wang T., Zhang X. (2024). A water-lean triazole ionic liquid biphasic solvent for energy-efficient CO_2_ capture through reversible polarity and intermolecular hydrogen bonding. Ind. Eng. Chem. Res..

[78] Arjona-Jaime P., Isaacs-Páez E.D., Nieto-Delgado C., Chazaro-Ruiz L.F., Rangel-Mendez R. (2024). Insight into the effect of pressure on the CO_2_ capture capacity and kinetics by a biochar-ionic liquid composite. J. Environ. Chem. Eng..

[79] Barrera Bogoya A., Arnal-Herault C., Barth D., Mutelet F., Belaissaoui B., Pinilla Monsalve L., Marchal P., Tamura Y., Nakama Y., Hayano S. (2024). CO_2_ sorption of elastomer poly(ionic liquid)s with imidazolium cations having different alkyl chains: Structure-morphology-property relationships and thermodynamic modelling by the PC-SAFT equation of state. Polymer.

[80] Barrulas R.V., Tinajero C., Ferreira D.P.N., Illanes-Bordomás C., Sans V., Carrott M.R., García-González C.A., Zanatta M., Corvo M.C. (2024). Poly(ionic liquid)-based aerogels for continuous-flow CO_2_ upcycling. J. CO2 Util..

[81] Chaouiki A., Chafiq M., Ko Y.G. (2024). Unveiling the mechanisms behind high CO_2_ adsorption by the selection of suitable ionic liquids incorporated into a ZIF-8 metal organic framework: A computational approach. Environ. Res..

[82] Duarte E., Bernard F., Dos Santos L.M., Polesso B.B., Duczinski R., Forneck V., Geshev J., Einloft S. (2024). CO_2_ capture using silica-immobilized dicationic ionic liquids with magnetic and non-magnetic properties. Heliyon.

[83] Fu M., Ding W., Zhao Q., Xu Z., Hua W., Li Y., Yang Z., Dong L., Su Q., Cheng W. (2024). Dual hydrogen bond donor functionalized hierarchical porous poly(ionic liquid)s for efficient CO_2_ fixation into cyclic carbonates. Sep. Purif. Technol..

[84] Guo C., Guo P., Zhou Y., Yan X., Li H., Liu D. (2024). A core-shell IL@MOF composite with ultra-high selectivity in multiple CO_2_ purification systems. Chem. Eng. Sci..

[85] Huang J., Wang J., Duan H., Dong L., Chen S., Zhang J., Zhang X. (2024). Zr modulated N doping composites for CO_2_ conversion into carbonates. iScience.

[86] Hussain S., Dong H., Duan H., Ji X., Asif H.M., Liu W., Zhang X. (2024). Efficient selective carbon dioxide separation via task-specific ionic liquids incorporated in ZIF-8. Langmuir.

[87] Jadav D., Pandey M., Bhojani A.K., Amen T.W.M., Tsunoji N., Singh D.K., Bandyopadhyay M. (2024). Mesoporous silica supported ionic liquid materials with high efficacy for CO_2_ adsorption studies. J. Ion. Liq..

[88] Kang D.A., Murphy C., Jeong H.-K. (2024). Improving CO_2_ adsorption capacity and CO_2_/N_2_ selectivity of UiO-66-NH_2_ via defect engineering and IL-encapsulation. Microporous Mesoporous Mater..

[89] Kuchekar S., Gaikwad S., Han S. (2024). Design and synthesis of Mg-HKUST-1/solid ionic liquid composites for CO_2_ capture with improved water stability. J. Environ. Chem. Eng..

[90] Li B., Zeng S., Jiang C., Li G., Bai L., Xu F., Zhang X. (2024). Dynamic breakthrough performance of low concentration CO_2_ adsorption in fixed-bed column with porous amino acid ionic liquid composites. Sep. Purif. Technol..

[91] Shi T., Zheng Y., Wang T., Li P., Wang Y., Yao D. (2018). Effect of pore size on the carbon dioxide adsorption behavior of porous liquids based on hollow silica. ChemPhysChem.

[92] Yin Z., Chen H., Yang L., Peng C., Qin Y., Wang T., Sun W., Wang C. (2021). Investigations of CO_2_ capture from gas mixtures using porous liquids. Langmuir.

[93] Liu S., Liu J., Hou X., Xu T., Tong J., Zhang J., Ye B., Liu B. (2018). Porous liquid: A stable ZIF-8 colloid in ionic liquid with permanent porosity. Langmuir.

[94] Bennett T.D., Coudert F.X., James S.L., Cooper A.I. (2021). The changing state of porous materials. Nat. Mater..

[95] Li Y., Yuan S. (2024). The role of the nanoconfinement effect in the adsorption of carbon dioxide by porous liquids. ACS Sustain. Chem. Eng..

[96] Liao Q., Yuan Y., Cao J. (2024). One-step synthesis of hydroxyl-functionalized ionic hyper-cross-linked polymers with high surface areas for efficient CO_2_ capture and fixation. J. Colloid Interface Sci..

[97] Philip F.A., Henni A. (2024). Functionalization of ordered mesoporous silica (MCM-48) with task-specific ionic liquid for enhanced carbon capture. Nanomaterials.

[98] Taylor C.D.L., Klemm A., Al-Mahbobi L., Bradford B.J., Gurkan B., Pentzer E.B. (2024). Ionic liquid-glycol mixtures for direct air capture of CO_2_: Decreased viscosity and mitigation of evaporation via encapsulation. ACS Sustain. Chem. Eng..

[99] Qu Q., Cheng L., Wang P., Fang C., Li H., Ding J., Wan H., Guan G. (2024). Guanidine-functionalized basic binuclear poly(ionic liquid)s for low partial pressure CO_2_ fixation into cyclic carbonate. Sep. Purif. Technol..

[100] Wang H., Gao Z., Sun J. (2024). The synthesis of amide bond by a simple one-step method to connect ionic liquid and MIL-101-NH2 firmly for efficient CO_2_ cycloaddition. Sep. Purif. Technol..

[101] Yang J., Gao D., Pan Y., Cao Y., Zhang H., Chen Y. (2024). Experimental nibble for computational chemists: On the construction and CO_2_ capture by different zeolite imidazole ester framework-8 and ionic. Int. J. Quantum Chem..

[102] Yang J., Gao D., Zhang H., Yi Q. (2024). Construction of ZIF-8 and amino functionalized porous ionic liquids for efficient CO_2_ capture. Fuel.

[103] Ye N., Shen Y., Chen Y., Cao J., Lu X., Ji X. (2024). Enhanced CO_2_ capture through SAPO-34 impregnated with ionic liquid. Langmuir.

[104] Zhang X., Lang B., Song D., Li Y. (2024). Enhancing CO_2_ adsorption capacity of hydroxypyridine-based ionic liquids using fluorinated graphene as carrier Material: A density functional theory study. Appl. Surf. Sci..

[105] Zhang Y., Xu H., Wu H., Shi L., Wang J., Yi Q. (2024). Controllable construction of ionic frameworks for multi-site synergetic enhancement of CO_2_ capture. Front. Chem. Sci. Eng..

[106] Alcantara M.L., de Almeida Ribeiro Oliveira G., Lião L.M., Ortiz A., Mattedi S. (2024). Highly selective CO_2_/CH_4_ membranes based on ethanolamine ionic liquids. Ind. Eng. Chem. Res..

[107] Chamoun-Farah A., Keller A.N., Balogun M.Y., Cañada L.M., Brennecke J.F., Freeman B.D. (2024). Amine functionalized supported ionic liquid membranes (SILMs) for CO_2_/N_2_ separation. J. Membr. Sci..

[108] Chang Q., Guo H., Shang Z., Zhang C., Zhang Y., Dong G., Shen B., Wang J., Zhang Y. (2024). PIM-based mixed matrix membranes containing covalent organic frameworks/ionic liquid composite materials for effective CO_2_/N_2_ separation. Sep. Purif. Technol..

[109] Ferrari H.Z., Bernard F., dos Santos L., Dias G., Le Roux C., Micoud P., Martin F., Einloft S. (2024). Enhancing CO_2_/N_2_ and CO_2_/CH_4_ separation in mixed matrix membrane: A comprehensive study on Pebax^®^1657 with SSMMP/IL for improved efficiency. Polym. Eng. Sci..

[110] He S., Kamio E., Matsuoka A., Nakagawa K., Yoshioka T., Matsuyama H. (2024). Development of a double-network ion gel membrane composed of a CO_2_-philic ionic liquid, semi-crystalline polymer network, and tetra-armed polyethylene glycol network. J. Membr. Sci..

[111] Kang M., Min H.J., Kim S.-J., Kim J.H. (2024). Thin-film composite mixed-matrix membrane based on polymerizable ionic liquid comb copolymer for CO_2_ separation. J. Membr. Sci..

[112] Kontos G., Tsioptsias C., Tsivintzelis I. (2024). Cellulose acetate–ionic liquid blends as potential polymers for efficient CO_2_ separation membranes. Polymers.

[113] Roppolo I., Zanatta M., Colucci G., Scipione R., Cameron J.M., Newton G.N., Sans V., Chiappone A. (2024). Digital light processing 3D printing of polymerizable ionic liquids towards carbon capture applications. React. Funct. Polym..

[114] Yu Y., Yang X., Zhang C., Chen J., Lin W., Meng J. (2024). A tough double-network ion gel membrane based on poly (ionic liquid) for efficient carbon capture. Sep. Purif. Technol..

[115] Zhang M., Chen L., Wang K., Lin R., Xiao Z., Semiat R., He X. (2024). Molecular engineering of copoly(ionic liquids)-based membranes for CO_2_ separation. ACS Appl. Polym. Mat..

[116] Zhang M., Semiat R., He X. (2024). Highly CO_2_-selective composite membranes from amino-functionalized imidazolium-based Poly(ionic liquids). Sep. Purif. Technol..

[117] Zhong S., Xiang Y., Dai Y., Wang Y., Su W., Li S., Li J. (2024). Construction of PEO@g-C_3_N_4_ supported ionic liquid membrane with interactive network structure for enhanced carbon capture efficiency. J. Membr. Sci..

